# The methane-oxidizing microbial communities of three maar lakes in tropical monsoon Asia

**DOI:** 10.3389/fmicb.2024.1410666

**Published:** 2024-07-09

**Authors:** Iona Eunice C. Bicaldo, Karol Sophia Agape R. Padilla, Tzu-Hsuan Tu, Wan Ting Chen, Milette U. Mendoza-Pascual, Carmela Vannette B. Vicera, Justine R. de Leon, Kamille N. Poblete, Eleanor S. Austria, Mark Louie D. Lopez, Yuki Kobayashi, Fuh-Kwo Shiah, Rey Donne S. Papa, Noboru Okuda, Pei-Ling Wang, Li-Hung Lin

**Affiliations:** ^1^The Graduate School, University of Santo Tomas, Manila, Philippines; ^2^Research Center for the Natural and Applied Sciences, University of Santo Tomas, Manila, Philippines; ^3^Philippine Genome Center, University of the Philippines, Quezon City, Philippines; ^4^Department of Science and Technology, Science Education Institute, Taguig, Philippines; ^5^Department of Geosciences, National Taiwan University, Taipei, Taiwan; ^6^Department of Oceanography, National Sun Yat-sen University, Kaohsiung, Taiwan; ^7^Department of Environmental Science, School of Science and Engineering, Ateneo Research Institute for Science and Engineering, Ateneo de Manila University, Quezon City, Philippines; ^8^Department of Biological Sciences, University of Santo Tomas, Manila, Philippines; ^9^Biology Department, Adamson University, Manila, Philippines; ^10^Department of Biochemistry and Microbiology, University of Victoria, Victoria, BC, Canada; ^11^Center for Ecological Research, Kyoto University, Shiga, Japan; ^12^Research Center for Environmental Changes, Academia Sinica, Taipei, Taiwan; ^13^Research Center for Inland Seas, Kobe University, Kobe, Japan; ^14^Research Institute for Humanity and Nature, Kamigamo Motoyama, Kita Ward, Kyoto, Japan; ^15^Institute of Oceanography, National Taiwan University, Taipei, Taiwan; ^16^Research Center for Future Earth, National Taiwan University, Taipei, Taiwan

**Keywords:** Proteobacteria, Verrucomicrobia, NC10, CARD-FISH, 16S rRNA gene, pmoA

## Abstract

Methane-oxidizing bacteria (MOB) is a group of planktonic microorganisms that use methane as their primary source of cellular energy. For tropical lakes in monsoon Asia, there is currently a knowledge gap on MOB community diversity and the factors influencing their abundance. Herewith, we present a preliminary assessment of the MOB communities in three maar lakes in tropical monsoon Asia using Catalyzed Reporter Deposition, Fluorescence In-Situ Hybridization (CARD-FISH), 16S rRNA amplicon sequencing, and pmoA gene sequencing. Correlation analysis between MOB abundances and lakes’ physicochemical parameters following seasonal monsoon events were performed to explain observed spatial and temporal patterns in MOB diversity. The CARD-FISH analyses detected the three MOB types (I, II, and NC10) which aligned with the results from 16S rRNA amplicons and pmoA gene sequencing. Among community members based on 16S rRNA genes, Proteobacterial Type I MOB (e.g., Methylococcaceae and Methylomonadaceae), Proteobacterial Type II (Methylocystaceae), Verrucomicrobial (Methylacidiphilaceae), Methylomirabilota/NC10 (Methylomirabilaceae), and archaeal ANME-1a were found to be the dominant methane-oxidizers in three maar lakes. Analysis of microbial diversity and distribution revealed that the community compositions in Lake Yambo vary with the seasons and are more distinct during the stratified period. Temperature, DO, and pH were significantly and inversely linked with type I MOB and Methylomirabilota during stratification. Only MOB type I was influenced by monsoon changes. This research sought to establish a baseline for the diversity and ecology of planktonic MOB in tropical monsoon Asia to better comprehend their contribution to the CH_4_ cycle in tropical freshwater ecosystems.

## Introduction

Methanotrophs or methane-oxidizing bacteria (MOB) are aerobic gram-negative bacteria that utilize methane (CH_4_) for carbon and energy. Due to its significant increase in production from both anthropogenic (i.e., fossil fuels, livestock, waste management, biomass combustion, rice agriculture, and biofuels) and natural sources, CH_4_ contributes to at least 15–20% of the Earth’s warming (Kirschke et al., 2013; Guerrero-Cruz et al., 2021). The majority of CH_4_ produced in lakes is by archaeal methanogens synthesized from organic matter in anoxic sediments, originating from the primary production catalyzed by both terrestrial and aquatic cyanobacteria. The total annual CH_4_ emissions from overall lakes amounts to at least 41.6 Tg, wherein high latitude lakes, temperate lakes, and subtropical/tropical lakes contribute to at least 9.4 Tg, 9.3 Tg, and 18.8 Tg of CH_4_ emissions annually, respectively (data exclusive of diffusion, ebullition, and turnover measurements) (Johnson et al., 2022). Current CH_4_ emission estimates in tropical lakes are still undervalued due to limited observational data, however, a pioneering study in the three maar lakes of the Philippines (Mendoza-Pascual et al., 2021) showed that profundal zones store high CH_4_ concentrations, which could potentially be emitted to the atmosphere due to lake mixing caused by typhoons and seasonal winds (Forster et al., 2007; Tsai et al., 2008). Researchers have recently concentrated on MOBs’ vital function in reducing CH_4_ emissions and studied their community compositions and influences in the past because they can oxidize CH_4_ in various conditions (Costello and Lidstrom, 1999; He et al., 2020).

MOB oxidizes CH_4_ when it diffuses into the water column during lake overturn, preventing CH_4_ emission from lakes (Bastviken et al., 2011; Esposito et al., 2023), which makes them essential mitigators of CH_4_ release from lakes’ deeper layers (Trotsenko and Murrell, 2008; Mayr et al., 2020; Ming et al., 2022). MOB communities also facilitate benthic-pelagic carbon intake and sequestration in aquatic food webs (Bastviken et al., 2003; Deines and Fink, 2011). Main MOB groups include those from the Proteobacteria phylum (*Gammaproteobacteria*: Type I, X and *Alphaproteobacteria*: Type II) that initiate CH_4_ oxidation through the particulate CH_4_ monooxygenase enzyme (pMMO) encoded by the conserved subunit pmoA (Dedysh et al., 2000; Dumont and Murrell, 2005; Theisen and Murrell, 2005; Vorobev et al., 2011; Kojima et al., 2014; Luke et al., 2014; Kalyuzhnaya et al., 2019). Aside from these, other MOBs from the Verrucomicrobial phylum (family Methylacidiphilaceae) are ubiquitous in acidophilic environments (Dedysh et al., 2021), while MOBs from the phylum NC10 (family Methylomirabilaceae) were mostly observed in anoxic and nitrite-reducing habitats due to their ability to catabolize CH_4_ through nitrite instead of oxygen (Kojima et al., 2014).

Despite several studies being conducted in different latitudinal zones, information regarding the environmental preferences of MOB and their abundance in tropical lakes is still lacking.

Temperature, pH, CH_4_, dissolved oxygen (DO), and nutrient load are the primary factors influencing MOB abundance as they are crucial for nutrient uptake (Tiwari et al., 2015; Zigah et al., 2015; Yang et al., 2019). Large reservoirs and deep lakes, like Lake Kivu, Lake Tanganyika, and Petit Saut Reservoir, are the only ones in the tropics where MOB studies have been undertaken (Dumestre et al., 2002; Durisch-Kaiser et al., 2011; Pasche et al., 2011; Zigah et al., 2015), and they await information from equally significant tropical countries, such as the Philippines. Most investigations from the said lakes used phospholipid fatty acid analysis (PLFA), flow cytometry, CARD-FISH, and 16S rRNA amplicon sequencing to pinpoint the aqueous column MOB communities, which showed type I and II MOB, and novel ANME being present in the nutrient and CH_4_-rich layer of Lake Kivu, while in Lake Tanganyika presence of type I and II MOB were also observed. As seasonal variation is also a potential factor in the distribution of MOB species, studies in the aforementioned reservoirs highlighted the significance of aerobic and anaerobic CH_4_ oxidation, with high aerobic oxidation during the dry season and AOM during the wet season. However, strong evidence or additional data to support these findings remain elusive. Due to a lack of information, it is vital to investigate the ecology and as the variety of the planktonic MOB community present in lakes in tropical monsoon Asia.

This study aims to present a preliminary assessment of the planktonic MOB communities in lakes within the tropical monsoon Asia region. We specifically aim to do this by using molecular techniques such as Catalyzed Reporter Deposition, Fluorescence In-Situ Hybridization (CARD- FISH), pmoA sequencing, and 16S rRNA amplicon sequencing. Here, the three maar lakes on Luzon Island in the Philippines (Lakes Yambo, Pandin, and Calibato) were selected as representative sites to demonstrate how the composition of tropical methanotrophic communities can vary seasonally (Southwest Monsoon - SW; Northeast Monsoon - NE). Different mixing regimes present in these crater lakes support distinct bacterial species that can produce or consume CH_4_ from the profundal zones and be emitted from the water column into the atmosphere. Using CARD-FISH, 16S rRNA gene and diversity analyses, pmoA gene sequencing for SW (stratification) and NE (mixing) episodes, this work attempts to build vertical depth profiles of the MOB community in the three maar lakes and therefore present a baseline data of these communities in Philippine lakes. Seasonal variation and several driving factors, such as temperature, pH, DO, chlorophyll a (Chl a), and CH_4_ concentrations, were taken into consideration in the research design to identify environmental parameters that drive the diversity and composition of MOB communities.

## Materials and methods

### Study area and sampling collection

**FIGURE 1:**
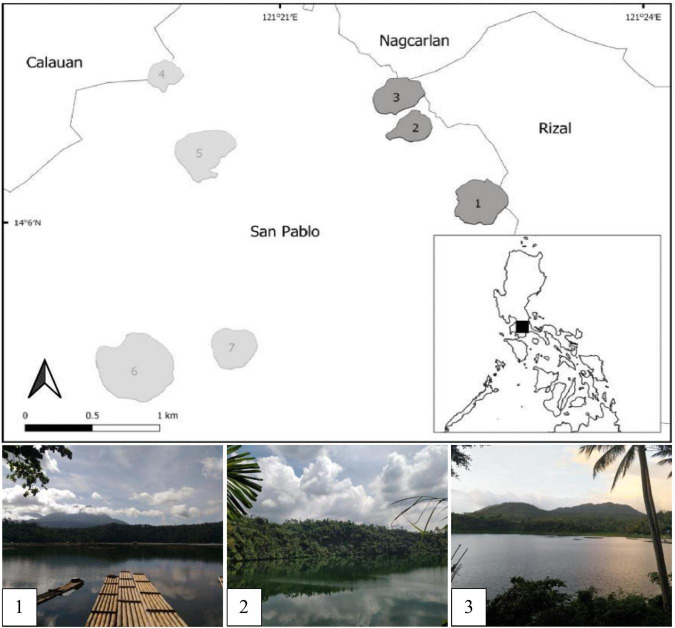
Map of the Seven Maar Lakes of San Pablo, Laguna, highlighting the three study sites. 1-Calibato, 2-Pandin, 3-Yambo, 4- Mohicap, 5-Palakpakin, 6-Sampaloc, and 7-Bunot.

The Seven Maar Lakes (SMLs) of San Pablo, Laguna are a collection of lakes that are believed to have formed as a result of the phreatic eruption of Mt. Banahaw-San Cristobal (LLDA, 2014; Bannister et al., 2019; Aguilar et al., 2023), and they are located in the southern region of Luzon Island, Philippines (Figure 1). These are categorized as small lakes (surface area ≤2,000,000 m^2^) impacted by a tropical monsoon climate (Brillo, 2016a,b; Aguilar et al., 2023) and having variable trophic statuses based on Chlorophyll a (Mendoza et al., 2019) and phytoplankton data (Navarrete et al., 2019). A simultaneous study with Aguilar et al. (2023) categorized the mixing patterns of the SMLs with Lakes Palakpakin, Bunot, Mohicap, Sampaloc, and Yambo being warm monomictic while the two deepest lakes Pandin and Calibato were categorized as meromictic. Thermal stratification of the lakes was observed during the SW monsoon from August 2018 and April and May 2022 while the complete mixing of the whole water column of Lake Yambo and the epilimnion of both Lakes Pandin and Calibato happened during the NE monsoon from November to February, which causes CH_4_ concentrations to be released from the water column into the atmosphere (Forster et al., 2007; Schubert et al., 2010; Mendoza et al., 2019; Mendoza-Pascual et al., 2021; Briddon et al., 2022). Lake Yambo, the deepest of the SML monomictic lakes, was selected as the primary study site to represent warm monomictic-mesotrophic lakes, while Lakes Pandin and Calibato, which are mesotrophic-meromictic and eutrophic-meromictic, respectively, were chosen as study sites to initially assess the diversity of the MOB community during the period of stratification (SW; August 2018) and mixing (NE; February 2019) (Table 1). After the preliminary assessment in 2018 and 2019, only Lake Yambo was monitored for MOB diversity in the SW (April and May) and NE (November and December) of 2022 (Table 1). Due to its similar mixing regime (monomictic; Aguilar et al., 2023) to lakes from temperate (Lake Biwa, Japan; Yoshimizu et al., 2010; Kojima et al., 2012) and subtropical (Fei Tsui Reservoir, Taiwan; Kobayashi et al., 2016) regions, Lake Yambo was also chosen as a representative site for tropical-region freshwater systems.

**TABLE 1:** Limnological characteristics of the three study sites, sampling coordinates, and sampling depths for both SW and NE months.

				Sampling depths (m) stratified period		Sampling depths (m) mixed period	
Lake	Lake surface area (m^2^)*	Maximum water depth (m)*	Sampling coordinates	Aug 2018	Sept, Nov, and Dec 2022	Feb 2019	Jan and Feb 2022
Yambo	305,000	33–36	14° 07′ 05.42′′ N 121° 22′ 01.63′′ E	0, 5, 15, 20, 25, 30, 35	0, 5, 15, 20, 25, 30, 32	5, 25, 35	5, 25, 32
Pandin	240,000	62	14° 06′ 54.79′′ N 121° 22′ 0.577′′ E	0, 5, 15, 30, 40, 50, 60		5, 15, 40, 60	
Calibato	430,000	131	14° 06′ 14.95′′ N 121° 22′ 38.68′′ E	0, 5, 15, 40, 60 80, 100, 125		5, 40, 80, 125	

*Data from LLDA, 2014.

An automated depth sounder (Hondex, Japan) was used to determine the maximum water depth. Temperature, DO, pH, and Chl a were measured using a multi-parameter vertical profiler (EXO 1-YSI, USA), while Mendoza-Pascual et al. (2021) simultaneously collected data on CH_4_ concentrations using the same methodology as Itoh et al. (2015), which was then used as supporting data in this study. Due to gas cylinder issues and failure to maintain the necessary consumable inventory for the gas chromatography in measuring the CH_4_ concentrations, Lake Yambo’s CH_4_ concentrations for the year 2022 were estimated using the Chl a data. As CH_4_ emitted from aquatic habitats exponentially increases with Chl a content in which plant-mediated transport is about 75% of total CH_4_ emissions, while ebullition accounted for 95%. (DelSontro et al., 2018; Beaulieu et al., 2019; Yang et al., 2022), the CH_4_ concentrations for the year 2022 were correlated and compared to the production data (Chl a) (Bastviken et al., 2004; Bastviken and Cole, 2008; Juutinen et al., 2009; West et al., 2016). Water samples for CARD-FISH, samples were in August 2018, February 2019, and 2022 (April, May, November, and December). The samples were subsequently treated with 2% (w/v) paraformaldehyde and filtered using a 0.20 μm pore- sized white polycarbonate membrane filter (Merck Millipore, Germany). Using 0.20 μm pore- sized cellulose nitrate membrane filters, water samples were collected for pmoA gene sequencing in February 2019 and for 16S rRNA gene sequencing in February 2019 and 2022 (September, November, and December).

### CARD-FISH for MOB abundance

Filtered samples for CARD-FISH analyses were processed following the method of Kobayashi et al. (2016), with some modifications from the work of Pernthaler et al. (2002). Filters were embedded in 0.01% low-melting point agarose (Amann and Fuchs, 2008) and permeabilized with lysozyme (Pavlekovic et al., 2009; Tischer et al., 2012; Kubota, 2013). Probes tagged with horseradish peroxidase, specific to detect the target MOB: type I (Mɣ705 and Mɣ84), type II (Mα450), and *M. oxyfera*-like organisms (DBACT-1027) were used (Eller et al., 2001; Heyer et al., 2002; Raghoebarsing et al., 2006; Kobayashi et al., 2016). Formamide concentrations for specific MOB probes include the following: Mɣ84 and Mɣ705 (20%), Mα450 (30%), and DBACT-1027 (55%). Signal amplification was initiated using Alexa Fluor 488 tyramide for 45 mins at 37°C. Filters were mounted on glass slides containing Vectashield (Vector Laboratories, USA) and 4′, 6-diamidino-2-phenylindole (DAPI). MOB counting for cell abundance was done for 10 fields of view for all depths and lakes under the Axioscope A1 epifluorescence microscope (Zeiss, Germany).

### 16S rRNA gene metabarcoding

Individually cut quarter-sized membranes were placed into tubes containing microbeads and lysis buffer for each depth collected. These mixtures were subjected to the bead-beating procedure for 2 min using a Tissue Lyzer (Natarajan et al., 2016; Manjarrés et al., 2022). While due to insufficient supply and equipment, the cells in 2022 were lysed using the modified bacterial lysis method of Martzy et al. (2019), which suspended the filtered cells in a TE buffer solution containing 1M Tris-HCl (pH 8.0), 0.1M EDTA, and 10% SDS (Wilson, 2001), and mixed using a vortex mixer at maximum speed for 10 min. Supernatants from each depth were placed into clean microtubes and processed using the DNeasy PowerLyzer PowerSoil Kit (Qiagen, USA) which has the same mechanism and principle as the method previously used. The QuantiFluor dsDNA System (Promega, USA) and NanoPhotometer, N60/N50 (Implen, Germany) were used for DNA quantification. The total cell concentrations at each depth were assessed to estimate the amount of DNA templates used in the polymerase chain reaction (PCR) amplification mix. The samples were amplified using primer pairs F515 (5′-GTGCCAGCMGCCGCGGTAA-3′) and R806 (5′-GGACTACHVGGGTWTCTAAT-3′) (Caporaso et al., 2011), which targets both archaeal and bacterial communities (Gulledge et al., 2001; Tu et al., 2017). DNA template (1–4 μl) was combined with 2 μl of forward and reverse primers, ex buffer, dNTP, Taq or 10 μl of Taq Master Mix, and 2 μl nuclease-free water for a total of 20–25 μl PCR reactions per depth. The PCR process was initiated for 2 min at 95°C followed by 30 cycles of 20 s at 95°C, 1 min at 55°C, and 1 min at 72°C to commence amplification. The final extension lasted for 10 min at 72°C. Successful PCR products (∼300 bp) were purified using a DNA Clean & Concentrator (Zymo Research, USA). A total of 60–70 μl of the purified sample was eluted. Sequencing of samples for 16S rRNA amplicons in 2019 and 2022 were carried out in Genomics Co., Ltd. (Taiwan) and Macrogen (Korea), respectively, using an Illumina sequencing platform.

### pmoA gene cloning and sequencing

pmoA genes were amplified with forward primer A189f which was paired with two other reverse primers, A650r (Bourne et al., 2001) and A621r (Tuomivirta et al., 2009). A total of 25 ul PCR reactions were prepared. PCR amplification was initiated with 3 mins of denaturation at 94°C, followed by 30 cycles of 30 s at 94°C, 45 s at 56°C, and 1 min at 72°C. Final extensions were made for 7 mins at 72°C. Gel extraction was performed following the manufacturer’s protocol for QIAquick Gel Extraction Kit (Qiagen, USA) and subjected to final purification using DNA Clean & Concentrator (Zymo Research, USA). Amplicon concentrations were determined using Quantifluor dsDNA System (Promega, USA). Ligation and transformation of pmoA gene amplicons were performed following the manufacturer’s protocol for pGEM-T Easy Vector System I (Promega, USA). At least 1 to 3ul of DNA templates were added to the ligation mix depending on the initial cell concentrations after the purification process. Successful transformants were mixed with JM109 High-Efficiency Competent Cells and streaked in individual LB agar plates with 20 mg/ml X-Gal Stock Solution, 100 mM (100×) of IPTG, and 100 mg/ml of ampicillin. At least 30 to 50 viable white colonies per depth were picked and re- incubated at LB broth containing the same components as mentioned above for at least 24 hours. Only viable reactions after the re-incubation process were processed for another round of PCR amplification using the same primer sets mentioned previously. Samples with the correct size (∼450 to 500 bp) were sent to Genomics Co. Ltd., Taiwan for sequencing.

### Data analyses

All 16S rRNA gene sequences were analyzed using Mothur v1.35 and Qiime2 following the protocol previously described by Estaki et al. (2020), Tu et al. (2017) and Schloss et al. (2009). Barcoded sequences were demultiplexed and low-quality reads (Phread score <25 and <33) were removed. The reads with more than two mismatches from the paired barcoded sequences were removed by noise reduction while maintaining the information from the representative reads and numbers of merged reads by pre-clustering and through the denoise_paired function in the Qiime2 (q2-dada2-plugin) (Huse et al., 2010; Callahan et al., 2016; Estaki et al., 2020). The unique reads were aligned with references to Silva NR132 SSU dataset and using QIIME 2 FeatureData[AlignedSequence] while the unaligned reads from the same region were removed and those sequenced regions beyond the primers were trimmed. Potential chimeric sequences were removed using the UCHIME program (Edgar et al., 2011). Sequencing sharing ≥97% identities were assembled into individual OTUs using the nearest neighbor joining algorithm (Schloss and Westcott, 2011; Yang et al., 2014). The sequencing (i.e., alpha rarefaction) depth was set to 30,000 based on the lowest number of sequences remaining in the sample after quality filtering and denoising (QIIME 2™) to estimate species richness. From the rarefied dataset, alpha diversity indices: number of observed OTUs, Chao-1, and Inverse Simpson indices (Chao, 1984; Faith, 1992) were computed. The Bray-Curtis dissimilarity matrix among samples was computed, and principal coordinate analysis (PCoA) diagrams were generated using Emperor in Qiime2 (Bray and Curtis, 1957). The association between categorical metadata columns and alpha diversity data was tested, and the Faith Phylogenetic Diversity was determined, a measure of community richness and evenness metrics. Sample composition was analyzed using Permutational multivariate analysis of variance (PERMANOVA) (Costello and Lidstrom, 1999; Anderson, 2001). Taxonomic classifications of each unique sequence were designated using the Silva NR132 SSU dataset, a pre-trained Naive Bayes classifier, and the q2-feature-classifier of the Qiime 2 plugin. The said classifier was trained on the Greengenes 13_8 99% OTUs, where the sequences were trimmed to include only 250 bases from the 16S region sequenced in this analysis (the V3 and V4 region, confined by the 515F/806R primer pair). Taxonomic designations with ≥80% bootstrap values were considered valid. Sequences sharing ≥97% identities were assembled into individual OTUs using the nearest neighbor algorithm (Schloss and Westcott, 2011; Yang et al., 2014). OTUs with families that are possible CH_4_-oxidizing organisms and methanogens were included in the generation of histograms using R script software and Sigma plot v11.

On the other hand, the pmoA sequences were trimmed for ambiguous ends, assembled, and categorized (OTUs at a 93% similarity cutoff), which corresponds to the 16S rRNA gene threshold of 97% similarity (Crevecoeur et al., 2017) using Sequencher 5.4.6 software. Without excluding the uncultured and cultured sample references from the database, the constructed OTUs were compared against the nucleotide BLAST database for sequence similarities. Both uncultured and cultured sequences with the highest similarities (>93% identity) from the three maar lakes samples were retrieved from the BLAST database and aligned using Muscle in MEGA v7 software. Maximum-likelihood trees were generated using 1000 bootstrap replicates using Hasegawa-Kishono-Yano (HKY) following the gamma-distribution model. All the sequences obtained from this study are available online at the NCBI database under BioProject PRJNA996764 with accession numbers SAMN36942344, SAMN36942413, SAMN36942414, SAMN36942426, SAMN36942427, SAMN36942471, SAMN36942474 for 16S rRNA gene and the pmoA gene sequences under BankIt 2770725 with GenBank accessions OR887221-OR887236.

MOB cell abundance was calculated using this formula:


c⁢e⁢l⁢l⁢a⁢b⁢u⁢n⁢d⁢a⁢n⁢c⁢e

$$\;=\frac{c\,o\,u\,n\,t\,s}{\left(1388*0.09843*0.0001\right)}*\left(1040*0.09843*0.0001\right)$$

$$\;*\left(\frac{n}{\left(1.25*1.25*3.14\right)}\right)$$

where,

counts = the number of total counts for the 10 fields of view

n = the amount of water sample (ml) passed through each filter (Kobayashi et al., 2016).

To determine the impact of the lakes’ physicochemical parameters on the MOB populations, Spearman rank correlation using R studio software was applied. While the comparison of the MOB communities within the three maar lakes was done using *T*-Test and Kruskal-Wallis analysis of variance using R studio.

## Results

### Lake physicochemical profiles

**FIGURE 2:**
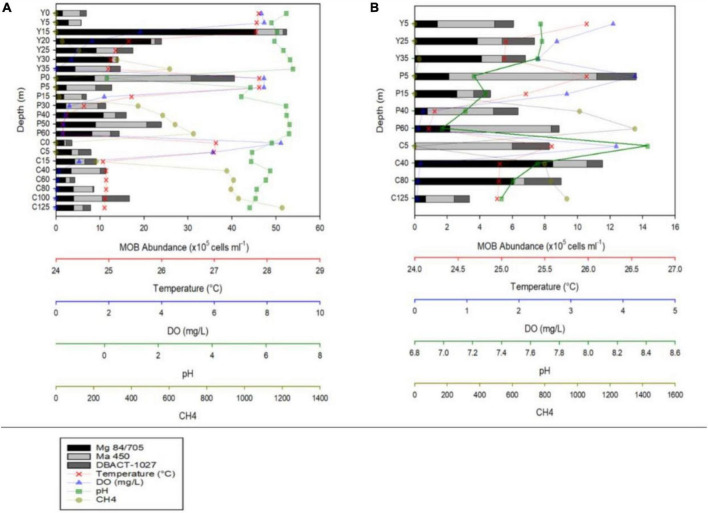
MOB abundances and vertical profiles of temperature, dissolved oxygen (DO), pH, and CH_4_ concentrations during the months of August 2018 **(A)** and February 2019 **(B)** of Lakes Yambo (Y), Pandin (P), and Calibato (C).

Lakes Yambo, Pandin, and Calibato exhibited average water temperatures (WT) of 26.28 ± 0.55°C, 25.44 ± 0.64°C, and 25.45 ± 0.34°C in SW August 2018 (Figure 2A and Supplementary Table 1). In NE February 2019, the three lakes experienced colder WTs than the SW (Figure 2B and Supplementary Table 1). The DO conditions were based on the standards of oxygenic levels used by Liu et al., 2019 and Aguilar et al., 2023, where the freshwater oxic is >4.0 mg/L, hypoxic is 2.0–4.0 mg/L, and anoxic is <2.0 mg/L. Lakes Yambo (0–20 m), Pandin (0–5 m), and Calibato (0–5 m) had oxic epilimnion layers with DO concentrations ranging from 3.19 to 8.52 mg/L during SW (Figure 2A and Supplementary Table 1). Their deeper layers were anoxic (0.00–1.83 mg/L). While DO concentrations from surface to deeper layers remained hypoxic to oxic throughout Lake Yambo (3.44–2.37 mg L^–1^), shallower epilimnion layers started to become anoxic in the metalimnion until near-bottom (0.40–0.07 mg L^–1^) in Lake Pandin and (0.49–0.05 mg L^–1^) Calibato in the NE (Figure 2B and Supplementary Table 1). However, near-bottom DO concentrations in Lake Calibato were anoxic and nearly undetectable (<0.05 mg L^–1^) (Figure 2B and Supplementary Table 1). The pH in all three lakes ranged from acidic to neutral (0.09–7.00) on SW (August 2018), while neutral to slightly alkaline (6.99 to 8.41) on NE (February 2019) (Figures 2A, B and Supplementary Table 1). CH_4_ concentrations were lowest (0.29–1.07 μmol L^–1^) in the surface depths (0 m) of all three lakes during stratification (SW) and increased with depth (Figure 2A). In contrast, CH_4_ concentrations were depleted (0.13–0.26 μmol L^–1^) in the 0–30 m of Lake Yambo during the NE, while the near-bottom depth (35 m) had a low concentration (28.17 μmol L^–1^) compared to the SW (378.44 μmol L^–1^). The deeper layers of Lakes Pandin and Calibato had higher CH_4_ concentrations in the NE than the SW. Despite being shallower (Pandin 62 m max, Calibato 131 m max; Table 1), Lake Pandin had greater CH_4_ concentrations (Figure 2B and Supplementary Table 1) than Calibato. During the NE, CH_4_ concentrations increased with depth in all three lakes, with the highest concentrations in near- bottom layers (Figure 2B and Supplementary Table 1).

**FIGURE 3:**
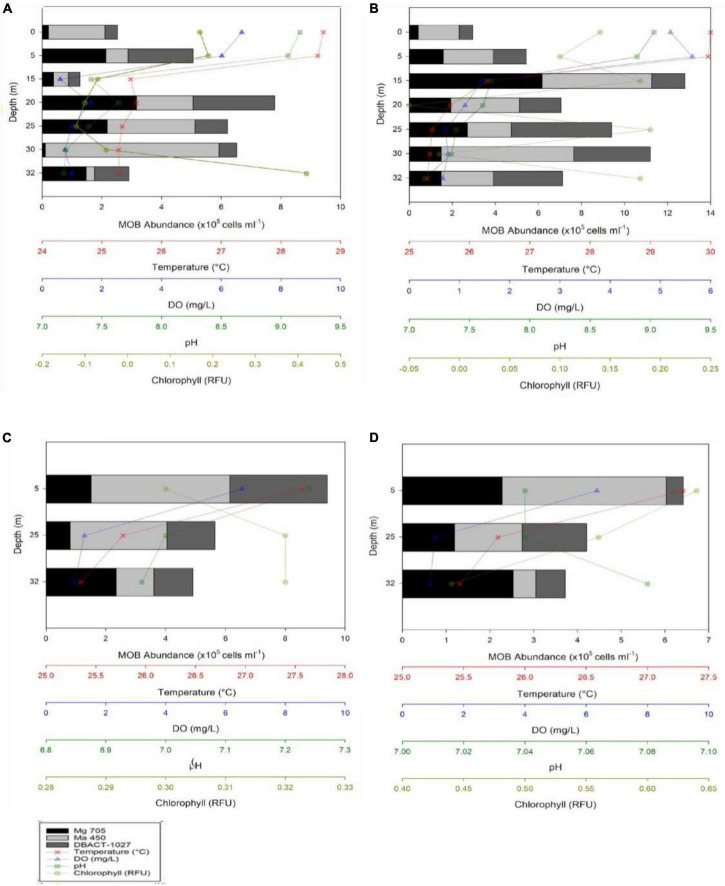
MOB abundances and Vertical depth profiles of temperature, dissolved oxygen (DO), pH, and chlorophyll concentrations during the months of May **(A)**, April **(B)**, November **(C)**, and December **(D)** 2022 in Lake Yambo.

In 2022, Lake Yambo continued to be monitored for its MOB community profiles. Lake Yambo’s WT average in the stratified months (April and May) were 26.33 ± 0.60°C and 26.85 ± 0.82°C, respectively (Figures 3A, B and Supplementary Table 1). In the NE mixed period (November and December), where stratification was weak, WT averaged 26.59 ± 0.39°C and 26.85 ± 0.82°C (Figures 3C, D and Supplementary Table 1). During the SW (April and May), Lake Yambo’s DO concentrations were still high (6.69–5.20 mg L^–1^) in the epilimnion (0–5 m) but became lower (0.60–1.63 mg L^–1^) from the metalimnion to the hypolimnion (15–32 m) (Figures 3A, B and Supplementary Table 1). Lake Yambo in the NE has oxic epilimnion and metalimnion (0–20 m; 6.56–2.28 mg L^–1^) and hypoxic hypolimnion (25–32 m; 1.28–0.91 mg L^–1^) (Figures 3C, D and Supplementary Table 1). The pH in SW (April and May) was basic (9.16–8.89) in the epilimnion (0–5 m) while neutral to slightly basic (7.68–7.13) in the metalimnion to hypolimnion (Figures 3A, B and Supplementary Table 1). In NE, neutral pH values were observed from 7.24 to 6.96 and 7.04 to 7.08 (Figures 3C, D and Supplementary Table 1). In the SW (April and May), the epilimnion (0–5 m) had 0.17–0.19 and 0.14–0.18 RFU, the middle layers (15–30 m) had −0.07 to −0.05 and −0.5 to −0.02 RFU, and the hypolimnion (32 m) had 0.42 RFU (Figures 3A, B and Supplementary Table 1). As the depth increased in the NE, Chl a concentrations increased from 0.30 to 0.32 RFU (November) and then decreased from 0.64 to 0.44 RFU in December (Figures 3C, D and Supplementary Table 1).

### CARD-FISH MOB abundance analysis

During the SW stratification (August 2018), CARD-FISH results in Lake Yambo showed the highest abundances of type I MOB in the epilimnion (15 m) with 45.46 × 10^5^ cells ml^–1^, type II in the same 15 m depth with 6.14 x10^5^ cells ml^–1^, and NC10 in the metalimnion (25 m) with 3.68 × 10^5^ cells ml^–1^. The sum of all three MOBs were the highest in the metalimnion (15–20 m) depths of Lake Yambo with 52.41 × 10^5^ cells ml^–1^ and 24.03 × 10^5^ cells ml^–1^, respectively (Figure 2A and Supplementary Table 2). In Lake Pandin, type I MOB was observed with the highest abundances in the deeper depths (40 m) (10.80 × 10^5^ cells ml^–1^), type II was present at all depths, with the highest concentrations at the surface (0 m) (21.93 × 10^5^ cells ml^–1^), and NC10 had its highest abundance at the surface (0 m) (9.82 × 10^5^ cells ml^–1^), and decreasing with depth. The sum of all three MOBs was at the maximum abundances at the surface (0 m) (40.55 × 10^5^ cells ml^–1^) and high at 40–50 m depths (16.00 × 10^5^ cells ml^–1^and 23.96 × 10^5^ cells ml^–1^) of Lake Pandin (Figure 2A and Supplementary Table 2). In the entire water column of Lake Calibato, all three MOB types were also found, but type I had relatively low abundances of about 1.89 × 10^5^ cells ml^–^1 to 4.31 × 10^5^ cells ml^–1^ in the epilimnion (0 to 15 m) and increased in number as the depth increased, with its highest abundance recorded at 100–125 m (4.07 × 10^5^ cells ml^–1^); type II had the highest abundance at 40 m (6.56 × 10^5^ cells ml^–1^) while NC10 had its highest abundance at 100 m (6.17 × 10^5^ cells ml^–1^) (Figure 2A and Supplementary Table 2). The sum of all three MOB types were high at 40 m (11.40 × 10^5^ cells ml^–1^) and 100 m (16.73 × 10^5^ cells ml^–1^) (Figure 2A and Supplementary Table 2). On the other hand, sampling depths for all three lakes were lessened during the NE mixing period (February 2019). Lake Yambo had all three MOBs during the NE, with highest abundance of type I at 35 m (4.10 × 10^5^ cells ml^–1^), type II at 5 m (3.51 × 10^5^ cells ml^–1^), and NC10 at 25 m (2.00 × 10^5^ cells ml^–1^) (Figure 2B and Supplementary Table 2). Lake Pandin also had substantial concentrations of all three MOBs in the water column: type I at 15 m (2.60 × 10^5^ cells ml^–1^), type II at 5 m (9.09 × 10^5^ cells ml^–1^), and NC10 at 5 m (2.42 × 10^5^ cells ml^–1^). Lake Calibato had high abundances at 40 m (8.49 × 10^5^ cells ml^–1^) for type I, 5 m (5.93 × 10^5^ cells ml^–1^) for type II and 5 and 80 m (2.28 × 10^5^ cells ml^–1^) for NC10 (Figure 2B and Supplementary Table 2).

**FIGURE 4:**
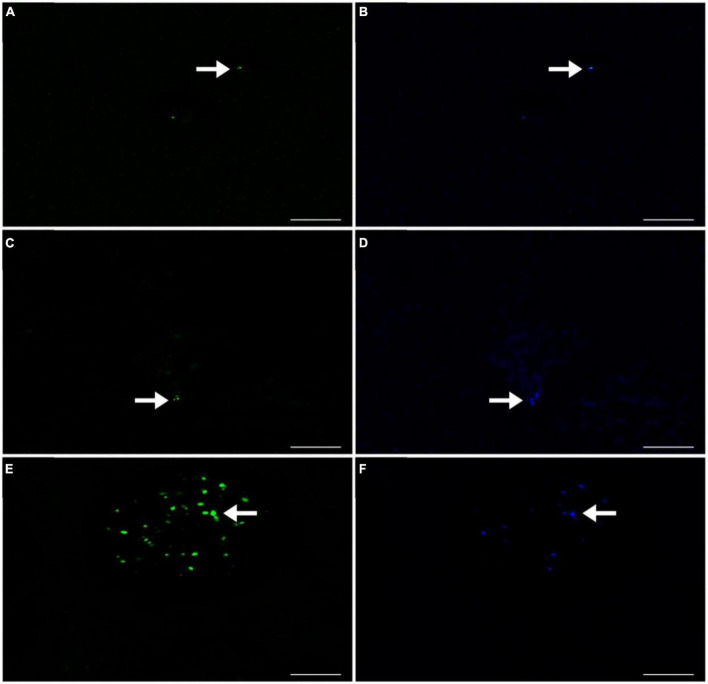
CARD-FISH Fluorescence images of the MOBs detected in Lake Yambo 2022. **(A)** Type I using Mγ705 probes labeled with Alexa Fluor™ 488 NHS Ester observed under FITC filter (green); **(B)** Type I using Mγ705 probes labeled with Alexa Fluor™ 488 NHS Ester observed under DAPI filter (blue); **(C)** Type II using Mα450 probes labeled with Alexa Fluor™ 488 NHS Ester observed under FITC filter (green); **(D)** Type II using Mα450 probes labeled with Alexa Fluor™ 488 NHS Ester observed under DAPI filter (blue); **(E)** NC10 using DBACT-1027 probes labeled with Alexa Fluor™ 488 NHS Ester observed under FITC filter (green); **(F)** NC10 using DBACT-1027 probes labeled with Alexa Fluor™ 488 NHS Ester observed under DAPI filter (blue). Scale bars represent 3 μm. Note: FITC - Fluorescein isothiocyanate filter (green) and DAPI - - 4’,6-diamidino-2-phenylindole filter (blue).

The presence of all three MOB types (Type I - Figure 4A, Type II - Figure 4C, and NC10 - Figure 4E) was observed in Lake Yambo for the 2022 monitoring, which was also supported by the total bacterial counts using DAPI (Figures 4B, D, E). During the SW (April and May) highest abundance of type I was observed at the metalimnion (20 m) with 3.16 × 10^5^ cells ml^–1^ and (15 m) (6.17 × 10^5^ cells ml^–1^) type II showed highest concentrations in the hypolimnion (30 m) with about 5.82 × 10^5^ cells ml^–1^ and 6.17 × 10^5^ cells ml^–1^; while NC10 had the highest abundance at the metalimnion depth (20 m) with 2.74 × 10^5^ cells ml^–1^ and (25 m) (4.67 × 10^5^ cells ml^–1^) (Figures 3A, B and Supplementary Table 2). Still, the highest abundances of MOB types are found in the metalimnion layer of Lake Yambo during the stratified period (April and May 2022) similar to the August 2018 data. During NE of November and December (2022) in Lake Yambo, the highest abundances of type I were observed at the hypolimnion layer (32 m) (2.35 × 10^5^ cells ml^–1^ and 2.53 × 10^5^ cells ml^–1^), type II at epilimnion (5 m) (4.63 × 10^5^ cells ml^–1^ and 3.75 × 10^5^ cells ml^–1^) and NC10 at the epilimnion (5 m) (9.40 × 10^5^ cells ml^–1^ and 6.42 × 10^5^ cells ml^–1^) (Figures 3C, D and Supplementary Table 2).

### 16S rRNA gene metabarcoding and bacterial diversity analysis

**FIGURE 5:**
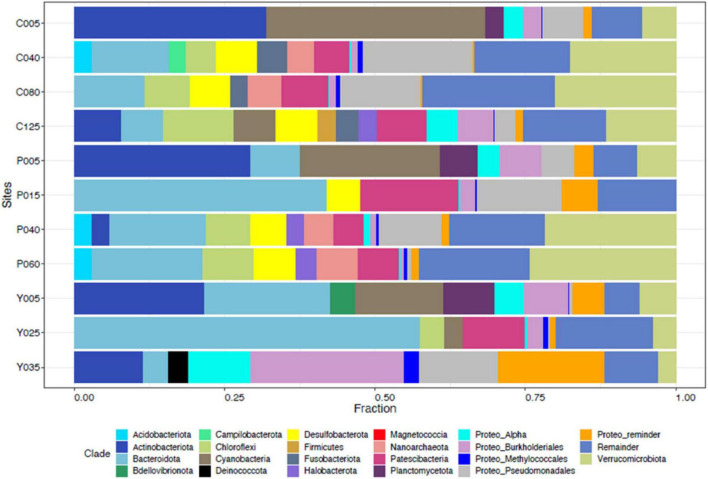
Taxonomic classification of the microbial communities in the Philippine maar lakes integrated from 16S rRNA Illumina analysis (2019). The *y*-axis represents the initial of each lake (C- Calibato, P- Pandin, and Y- Yambo) with the corresponding depths sampled.

**FIGURE 6:**
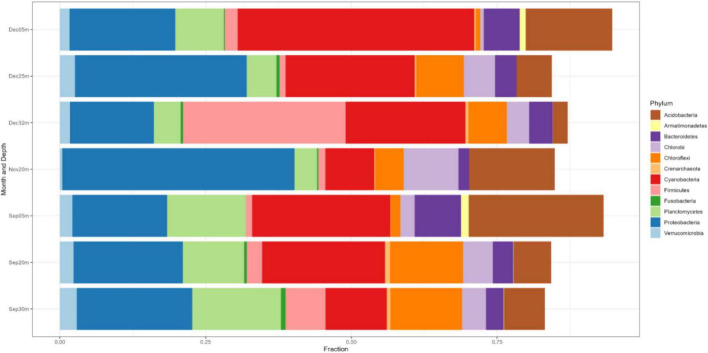
Phylum-level classification of the microbial communities in Lake Yambo during the SW stratification (September) and NE mixing (November and December) 2022 integrated from 16S rRNA analysis. The *y*-axis represents the sampling depths and months with the corresponding fractions of the identified species (*x*-axis).

In NE mixing of 2019, a total of 5,313 OTUs were obtained from the ≥6,000 reads per depth of the 16S rRNA gene dataset. Generally, the water column of the three lakes was mainly dominated by the following phyla: Actinobacteriota, Bacteroidota, Patescibacteria, Verrucomicrobiota, Chloroflexi, Desulfobacteriota, and several groups of Proteobacteria (Alpha- and Pesudomonadales) (Figure 5). Groups such as Cyanobacteria and Planctomycetes dominated the epilimnion (5 m) depths of the three lakes, while those from the phylum Halobacterota dominated the deeper depths of lakes Pandin (40 and 60 m) and Calibato (125 m) (Figure 5). In 2022, the 16S rRNA gene analyses in Lake Yambo for the seven (7) depths resulted in around 597,000 total paired reads. A similar trend from 2019 was observed in the epilimnion (5 m) depth of this lake for 2022 wherein Cyanobacteria, Planctomycetes, Actinobacteria, and Bacteroidota were the dominant groups (Figure 6). At the metalimnion layer (20–25 m), Cyanobacteria, Chloroflexi, Plactomycetes, and Chlorobiota were common. Whereas in the deepest depths (30–32 m), Firmicutes, Chloroflexi, Planctomycetes, and Cyanobacteria were the most common groups. Verrucomicrobia were also detected in these depths albeit with a lower frequency.

Generally, different groups of Proteobacteria (*Alpha-*, *Beta*-, *Gamma*-, and *Delta-*) were also present in all the months sampled for all depths (Figure 6). The relative frequency of the Alpha- and Beta- Proteobacteria was higher in the upper depths (5–20 m), while Gammaproteobacteria showed higher frequencies in the 20–32 m depths, especially during NE mixing (November and December) (Figure 6).

**FIGURE 7:**
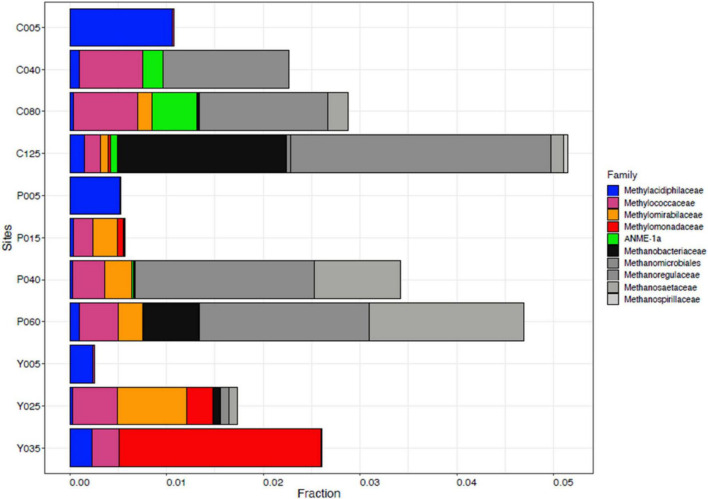
Family-level classification of MOB (colorful) and methanogens (grayscale) in Philippine maar lakes in the year 2019 integrated from 16S rRNA analysis. The *y*-axis represents the sampling sites (C-Calibato, P-Pandin, and Y-Yambo) with the corresponding depths sampled.

**FIGURE 8:**
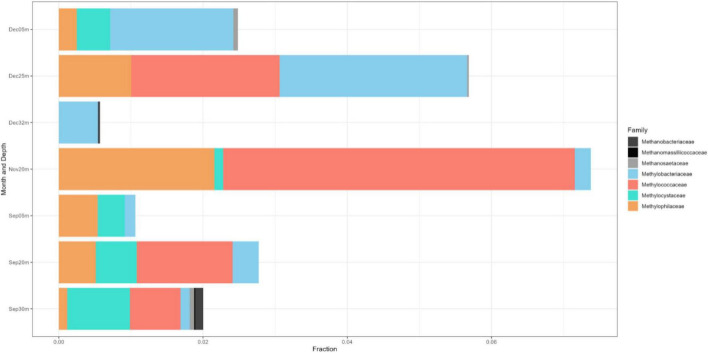
Family-level classification of the MOB (colorful) and methanogenic (grayscale) communities in Lake Yambo during the SW stratification (September) and NE mixing (November and December) 2022 integrated from 16S rRNA. The *x*-axis represents the corresponding number of sequencing reads and the *y*-axis represents the sampling months and depths.

The 16S rRNA gene dataset was further analyzed to enumerate which CH_4_-oxidizing (MO) organisms were present in the three lakes during NE mixing of 2019. Generally, the MOBs seen in almost all depths of the three lakes include the type I MOB families (and genera enclosed in parentheses) such as Methylococcaceae (*Methyloparacoccus*, *Methylogaea*, *Methylocaldum*, and uncharacterized genera) and Methylomonadaceae (*Methyloglobulus*, *Methylomonas*, and *Methylovulum*) (Figure 7). Other MOB families, such as Methylomirabilaceae from Methylomirabilota phylum (previously NC10 phylum; Bowman, 2014; Zhu et al., 2022; Figure 7 and Supplementary Table 5), with uncultured genera (Sh765B-TzT-35 to Sh765B-TzT-38) were also seen even in the oxic depth (25 m) of lake Yambo, and deeper depths of lakes Pandin (15, 40, and 60 m) and Calibato (80 and 125 m). Uncharacterized MOBs from the Methylacidiphilaceae family of the Verrucomicrobiota phylum were mostly dominant in the epilimnion depths (5 m) of the three lakes, albeit generally present in other depths of the three lakes as well (Supplementary Table 5). Archaeal ANME-1a from the Halobacterota phylum was also seen in the deeper layers of lakes Pandin (15 and 40 m) and Calibato (40, 80, and 125 m) (Figure 7 and Supplementary Table 5). MOB in Lake Yambo year 2022, composed of Proteobacterial type I families Methylococcaceae (*Methylomonas* and *Methylocaldum*) which is the same genera identified in 2019, and type II Methylocystaceae (*Methylosinus*), which was not observed in the dataset from 2019. This MOB was mainly found in the bottom depths (20 and 30 m) of Lake Yambo during the SW stratification (September) but was noted to be lesser in number during the NE transition (November) and mixing (December) (Figure 8). Presence of Proteobacterial methylotroph families or those capable of utilizing methyl-compounds like Methylobacteriaceae (*Methylobacterium* and an uncharacterized genus) and Methylophilaceae (uncharacterized genera) (Figure 8 and Supplementary Table 8) was also observed. Other MOB families like Methylomirabilaceae, Methylacidiphilaceae, and ANME-1a were not seen in any of the months sampled in 2022 for Lake Yambo. Families and genera such as Methanobacteriaceae (*Methanobacterium*), Methanoregulaceae (*Methanoregula* and *Methanolinea*), Methanocellales, Methanomassiliicoccaceae, Methanosaetaceae (*Methanosaeta*), and Methanospirillaceae (*Methanospirillum*) were detected in the three lakes during NE 2019 (Figure 7). Almost all of the species of these methanogens were uncharacterized in nature with respect to the polyphasic approach, and their presence was still noted even in the oxic depths (25 and 35 m) of Lake Yambo, while their highest fractions were seen in the deepest depths of lakes Pandin and Calibato (Figure 7 and Supplementary Table 5). In 2022 SW stratification (September), the presence of *Methanobacterium*, *Methanosaeta*, and ambiguous taxa of Methanomassiliicoccaceae was apparent in the 30 m depth of Lake Yambo, while their presence was also observed in the NE mixing (November and December) albeit in small abundances even in the oxic depths (5 m) as well as the other depths sampled for this lake (Figure 8 and Supplementary Table 8).

On the other hand, the microbial diversity in Lake Yambo was also assessed as the monitoring continued in 2022. As depicted in Supplementary Figure 1A, the Shannon index showed that the stratified month of September (∼7.5) has higher bacterial diversity richness than the mixing months of November (∼ 6.8) and December (∼ 6.5). Pielou’s evenness (Supplementary Figure 1B) in stratified conditions (September) (a median value of about 0.92) is higher than in mixed conditions (December) of about 0.81 Pielou index value (median). However, the November samples could not show the variation in species diversity and richness because it only has one sampling depth. The Pielou’s evenness by depth showed that 5 m samples (∼0.87 median Pielou index) have a relatively high proportion of bacterial species present compared to 20 and 30 m (∼0.84 - 0.85 median Pielou index). Although bacterial communities in 30 m have lower counts of species than in 5 m, the median is in the exact center of the 30 m-box plot, indicating that species are evenly distributed at this particular depth in Lake Yambo based on the 16S rRNA analysis.

**FIGURE 9:**
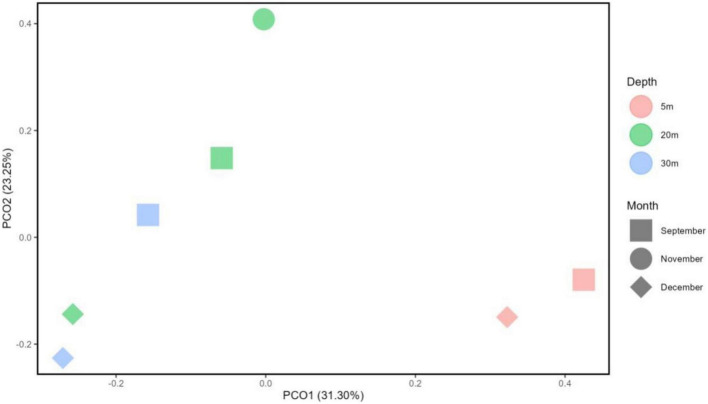
Principal component analysis (PCA) diagram using Bray-Curtis reflecting distances on the microbial OTU abundance between selected months and depths in Lake Yambo (2022).

Principal Coordinate Analysis (PCoA) based on Bray Curtis dissimilarity metrics (Figure 9) revealed that the metalimnion to hypolimnion layers (20–32 m) of September and December, as well as the epilimnion layer (5 m) of both months, exhibited close distances or similarities in their community composition. It is noted that the November metalimnion (20 m) has a different bacterial community composition because it was the transition month of Lake Yambo showing a weakly stratified vertical column (Figures 3C, 9).

### Phylogenetic analyses of pmoA gene clones

**FIGURE 10:**
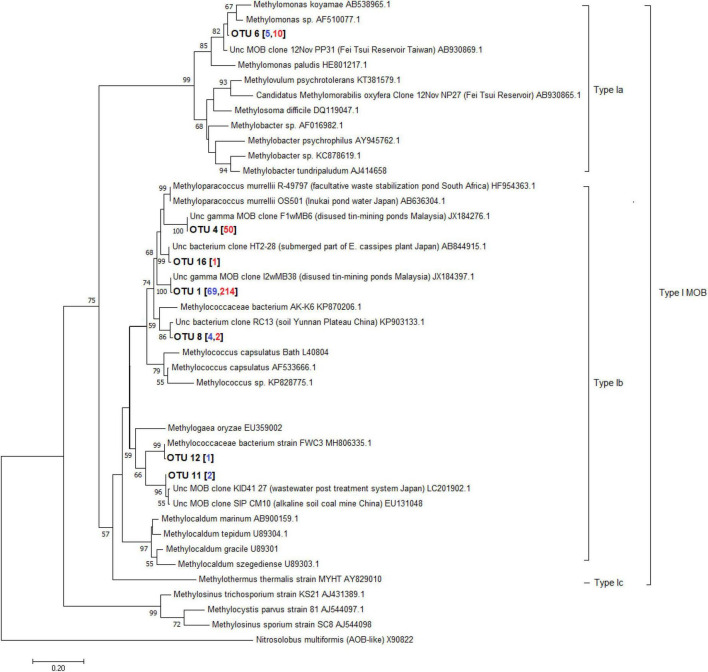
Maximum-likelihood tree of type I pmoA sequences constructed using 1000 bootstrap replicates. OTUs represent the sequences obtained from the three maar lakes of the Philippines. Numbers written in between the node branches represent bootstrap values (≥50). Numbers inside the parenthesis indicate the total sequences obtained in each primer set (blue – primers A189f/621r; red- primers A189f/650r).

**FIGURE 11:**
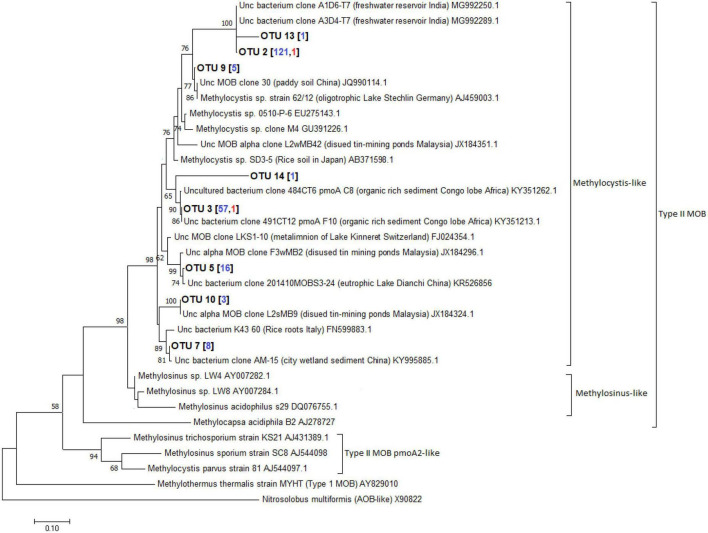
Maximum-likelihood tree of type II pmoA sequences constructed using 1000 bootstrap replicates. OTUs represent the sequences obtained from the three maar lakes of the Philippines. Numbers written in between the node branches represent bootstrap values (≥50). Numbers inside the parenthesis indicate the total sequences obtained in each primer set (blue – primers A189f/621r; red- primers A189f/650r).

A total of 598 sequences of the pmoA genes from the different depths of the three lakes constructed using primer combinations A189f/621r and A189f/650r were obtained. These sequences were grouped into 16 distinct OTUs, eight of which belonged to the type I MOB clade while the remaining eight belonged to the type II MOB lineage. Without the exclusion of uncultured MOBs using the mega blast search, OTUs from the Philippine maar lakes were highly identical (≥93%) to uncultured MOBs obtained from other terrestrial and aquatic environments (Supplementary Table 7). Phylogenetic analyses of the maximum-likelihood trees support their close relativity (≥65 bootstrap percent) with these uncultured MOBs. The largest total sequences and OTUs in the three lakes appear to be closely related to sequences from the type Ib genera *Methyloparacoccus, Methylococcus, Methylogaea*, and *Methylococcaceae* bacterium, while only OTU 6 belonged to the type Ia *Methylomonas*-like genera (Figure 10, Supplementary Figures 2–4, and Supplementary Table 7). On the other hand, the type II OTUs from the maar lakes appear to be closely related to the *Methylocystis*-like genera (Figure 11, Supplementary Figures 2–4, and Supplementary Table 7). Some of the OTUs for type I MOB (OTU 6 and OTU 11) and type II (OTU 9 and OTU 14) have no immediate close relation with the other uncultured and cultured MOBs (Figures 10, 11), probably representing novel groups of MOBs within the type I *Methylomonas*, *Methylococcaceae* bacterium, and type II *Methylocystis* lineage (Figures 10, 11, Supplementary Figures 2–4, and Supplementary Table 7).

### Correlation analysis of physicochemical parameters and MOB communities

Spearman’s rank correlation showed that temperature and pH were substantially associated with type I MOB abundance from CARD-FISH data in Lake Calibato during SW stratification (August 2018) with coefficients (*p*-values) of −0.87 (0.0048) and −0.85 (0.0075), respectively (Supplementary Table 3). Physicochemical parameters and the three types of MOBs were not correlated in the NE (February 2019). In the SW (May 2022), temperature, DO, and pH were substantially associated with NC10 abundances from CARD-FISH data in Lake Yambo with coefficients (*p*-values) of −0.85 (0.0162), −0.83 (0.0212), and −0.85 (Supplementary Table 3). Likewise, all physicochemical variables and the three MOB types did not correlate in NE (November and December 2022) (Supplementary Table 3).

Moreover, the *t*-test showed that type I MOB populations were significantly different within the three maar lakes with respect to the month (August 2018 and February 2019), *p*-value = 0.04198 (Supplementary Table 4). The Kruskal-Wallis Test for Lake Yambo demonstrated that type I MOB differs also by month in 2018–2019 and 2022, *p*-value = 0.04285 (Supplementary Table 4). Nonetheless, type II MOB and NC10 populations have no significant difference in both the depths and months among the three maar lakes studied, thereby connoting that the population medians for the said MOB group are equal.

## Discussion

### MOB communities and comparison with other regions in the world

Temperate lakes worldwide were studied for their MOB (Methanotrophic Bacteria) community organization. In Lake Mizugaki, Japan, type I MOBs, particularly *Methylobacter*, dominated the stratified water column, while in oligotrophic Lake Constance, Germany, they outnumbered type II (Kojima et al., 2009; Oswald et al., 2015). Similarly, three maar lakes investigated showed prevalent type I and II MOBs throughout the water column. Specifically, Gammaproteobacterial type I MOB of the Methylococcaceae family (*Methyloparacoccus, Methylogaea, Methylocaldum*, and uncultured Methylococcaceae bacterium) and Methylomonadaceae family (*Methyloglobulus, Methylomonas, and Methylovulum*) were dominant in all three maar lakes (Figures 7, 8 and Supplementary Tables 5, 7, 8).

Anoxic environments favored type I *Methylobacter* clones (Blees et al., 2014). In the eutrophic sub-alpine monomictic Lake Rotsee, type I *Methylomonas* were the most active MOBs oxidizing CH_4_. Type I MOB oxidized CH_4_ in oxygen-depleted Lake Cadagno (Dumestre et al., 2002; Milucka et al., 2015). These studies show that type I MOB *Methylobacter* oxidizes CH_4_ in both oxic and anoxic temperate lakes, with NC10 as the major oxidizer. Subtropical MOB community assemblage study focused on reservoirs. 16S rRNA gene cloning and CARD-FISH found many genera of type I MOB, type II (*Methylocystis*), and NC10 Ca. *Methylomirabilis oxyfera* in Fei Tsui Reservoir (Taiwan) (Kojima et al., 2014). The novel type Ia MOBs from maar lakes were closely linked to uncultured *Methylomonas* from Fei-Tsui Reservoir in Taiwan (Figure 10, Supplementary Figures 2–4, and Supplementary Table 7), along with NC10 Ca. *Methylomirabilis oxyfera* in its anoxic stratum. Kobayashi et al. (2016) found type II MOB in all depths and type I and NC10 in oxygen-depleted waters in the reservoir’s vertical distribution and seasonal profile. Type II *Methylocystis*-like MOBs from freshwater reservoirs in India, organic-rich sediment in Congo lobe Africa, eutrophic lakes in China, and Malaysian disused tin-mining ponds were similar to those in the sampled lakes (Figure 11, Supplementary Figures 2–4, and Supplementary Tables 7, 8; Roland et al., 2018).

In 2022, 16S rRNA gene metabarcoding using the same primer revealed type II MOB from the Methylocystaceae family (especially *Methylosinus*) in Lake Yambo (Figure 8 and Supplementary Table 8), verified by CARD-FISH (Figures 3, 4 and Supplementary Table 2). Alphaproteobacteria were discovered at varied depths, but their sequencing provided uncultured or methylotrophic species (i.e., those utilizing single-carbon compounds such as *Methylobacterium*, Methylophilaceae) (Figures 6, 8 and Supplementary Table 8). *Methylobacterium* was reported to use CH_4_ by Van Aken et al. (2004), but recent experiments by Iguchi et al. (2014) and Green and Ardley (2018) showed that the whole genus does not use CH_4_ and does not contain the enzyme for CH_4_ oxidation (*pMMO*), suggesting that it is not a MOB. The absence of the Methylocystaceae type II family from the 2019 Illumina 16S rRNA gene study may be due to the low detection of this taxon using the 16S primers and without incorporating other conserved variable regions that distinguish higher-ranking taxa. Tu et al. (2017) and Lin et al. (2018) detected Alphaproteobacteria but not Methylocystaceae using the same 16S rRNA primer set.

This research conferred on details regarding the diversity and ecological behavior of poorly studied MOBs in Philippine maar lakes, emphasizing their importance in reducing greenhouse gas emissions and regulating carbon cycling in aquatic environments. Moreover, methanogens in the deeper water column of the three maar lakes suggest CH_4_ generation is not limited to silt with high CH_4_ concentrations. Furthermore, studies found various microbial consortia in aquatic environments (Vuillemin et al., 2017), but none explored the tropical maar lake MOB community structure.

### Driving factors of diversity for CH_4_-oxidizing organisms in tropical monsoon Asia

Currently, the primary drivers for MOB community proliferation are not yet clearly established, but since the process of methanogenesis is dependent on factors such as temperature, available vegetation, nutrients, and organic material present (Marinho et al., 2009; Chang et al., 2010), these could also be the factors affecting MOB growth in these habitats as they directly increase CH_4_ production (Borjesson et al., 2004; Singh, 2013; Yvon-Duroucher et al., 2014; Zigah et al., 2015; Rybak et al., 2018). Temperature and pH have a significant and negative relationship with type I MOB during the SW stratification (August 2018) in Lake Calibato (Supplementary Table 3). In the SW stratification (May 2022), NC10 was observed to have a significant and negative correlation with temperature, DO, and pH. In previous studies, methanotrophic activity associated with types I and II metabolisms was found to be connected with temperature ranges (Trotsenko and Murrell, 2008; Roldán et al., 2022) where type I MOB is prevalent at psychrophilic temperatures (0–10 °C), while type II MOB are most prevalent at mesophilic temperatures (20–45°C) (Urmann et al., 2009; Graef et al., 2011; Roldán et al., 2022).

Correlation analyses conducted in 2022 for Lake Yambo showed that none of the parameters had a significant relationship with both Proteobacterial MOBs during the SW and NE monsoons.

Thermal stratification can cause anoxia to form in the bottom waters, allowing significant quantities of CH_4_ to accumulate (Schubert et al., 2012). During this phase, the highest MOB activity is found at the base of the oxycline, where the CH_4_ and DO counter gradients meet (Bastviken et al., 2002; Sundh et al., 2005; Bastviken and Cole, 2008; Borrel et al., 2011). As stratification progresses, these CH_4_-oxidation zones migrate within the water column (Carini et al., 2005; Guggenheim et al., 2020; Saarela et al., 2020). Similar to the 2018–2019 findings, MOB abundance is low during the mixing period because of the overturn and lower CH_4_ concentrations available in the water column as it is advected into the atmosphere (Costello et al., 2002; Lambrecht et al., 2019). Additionally, weak stratification and breaking of the thermocline (De Leon et al., 2024) during NE (November and December) 2022 could possibly have contributed to the insignificant correlation of all three MOB types.

Thus, MOB community composition and dominance vary during the mixing and transition seasons due to fluctuating nutrient availability and metabolic budgets (Kaupper et al., 2021; Merino-Ibarra et al., 2021). In the three maar lakes sampled, type I MOB was found to be abundant in the oxic-anoxic regions which could be due to high-affinity cytochromes (e.g., cytochrome-*bd*-ubiquinol- oxidoreductase) that assist CH_4_ oxidation under low DO concentrations (nM range) (Smith and Wrighton, 2019). Type II MOB, on the other hand, was mostly found in the oxic epilimnion (Supplementary Table 2) during lake overturn.

The 16S rRNA results for 2019 coincided with Naqvi et al. (2018) study, wherein Verrucomicrobial Methylacidiphilales (referred to as type III in their study) were dominant in the oxic upper depths of Indian dam reservoirs. Given the average temperature (25.42°C) and pH (6.73) in the three Philippine lakes sampled, which are not within the range of their optimal growth from studies (35–44°C and ph < 2.5) (Bodrossy et al., 1997; Dunfield et al., 2007; Pol et al., 2007; Van Teeseling et al., 2014), the Methylacidiphilaceae were still seen in all the depths of the lakes (Figure 7 and Supplementary Table 5). Methylacidiphilaceae also has a high affinity for hydrogen gas (H_2_), which allows them to thrive in hostile volcanic environments (Mohammadi et al., 2019). Culture experiments by Gagliano et al. (2014) from volcanic geothermal soils in Italy showed uncharacterized Verrucomicrobial MOB having the same pH and temperature as the three maar lakes. Since the lakes in this study formed from the phreatic eruption of a nearby volcano, the MOB communities present could be more similar to those MOBs found in geothermal environments, albeit novel due to their non-classification into certain genera of the Methylacidiphilaceae family. Interestingly, three distinct groups of the archaeal ANME-1a were also present in the hypoxic-and anoxic depths of lakes Pandin and Calibato (Figure 7 and Supplementary Table 5), supporting the notion that not only Proteobacterial MOBs are the active MO organisms in lake ecosystems (Eller et al., 2005). These groups are frequently found in a syntrophic relationship with sulfate-reducing bacteria (Boetius et al., 2000; Orphan et al., 2001; Michaelis et al., 2002). Despite being sensitive to DO fluxes (Knittel et al., 2005) and having low affinity for CH_4_ concentrations (Blumenberg et al., 2004), ANME-1a was still seen in the two-deep lakes in this study. Lastly, MOB from the phylum Methylomirabilota (NC10) were both detected in the three lakes using CARD-FISH and 16S rRNA gene metabarcoding. This phylum is known to associate denitrification with anaerobic methanotrophy (Deutzmann and Schink, 2011; He et al., 2016; Graf et al., 2018). Its unique ability to oxidize CH_4_ directly from nitrite reduction allowed its proliferation in anoxic conditions in freshwater sediments (Raghoebarsing et al., 2006; Ettwig et al., 2009; Shen et al., 2012). Even though *Methylomirabilis* sp. shares metabolic characteristics with proteobacterial MOBs, it belongs to the novel phylum NC10 which mediates the anaerobic oxidation of CH_4_ (AOM) (Holmes et al., 2001; Rappé and Giovannoni, 2003; Van Grinsven et al., 2021). CARD- FISH conducted in this study confirmed the presence of NC10 in all the layers but with the highest quantities in the deeper depths, especially during SW stratification (Figures 2A, 3A, B). In previous studies, Methylomirabilota or NC10 in some studies were noted to be sensitive to DO fluxes, in which an increase of at least 2–8% in oxygen reduced the rate of conversion by Methylomirabilota for CH_4_ and nitrite (Luesken et al., 2012), while its exposure to increased DO levels showed oxidative stress despite its oxygenic capacity (Luesken et al., 2012). Correlation analysis in this study showed that the Methylomirabilota MOB were significantly and inversely influenced by temperature, DO, and pH during the SW stratification of 2022 (Supplementary Table 3), which supports the established ranges from studies showing that Methylomirabilota proliferates in environments with pH 5-9 (Zhu et al., 2015; Welte et al., 2016). Since the anaerobic oxidation processes happen in most acidic environments, Methylomirabilota phylum also favors low pH conditions, as seen in the study, avoiding the increasing pH in the upper depths of the lakes (Figures 2, 3 and Supplementary Table 1).

The identified MOB species of the stratified month (September 2022) were observed to be more distinct and have higher sequence reads than the mixed month (December 2022) (Figure 8 and Supplementary Table 8). The frequency of methanogenic communities in Lake Yambo is also declining with the shift to the NE monsoon (Figure 8). Another probable reason for the decline in MOBs in Lake Yambo during the NE monsoon is that there is higher CH_4_ emitted to the atmosphere due to the lake’s complete mixing, thereby limiting the supply of carbon for MOBs’ growth and proliferation in the vertical water column (Mendoza et al., 2019; Mendoza-Pascual et al., 2021). Moreover, the three maar lakes assessed in this study for MOB community composition showed that type I MOB is significantly affected by seasons (Supplementary Table 4). The comparison of 2018–2019 and 2022 MOB in Lake Yambo also showed that type I MOB abundance responds to changing periods. Based on the analysis, Type I abundance changed over time (Kruskal-Wallis, *p* = 0.04285) and this may be due to the changes induced by different monsoons in the Philippines (Supplementary Table 4). Type II methanotrophs are adapted to oligotrophic and fluctuating environments, but their growth rates are low in these conditions, making them K-selected organisms (Shiau et al., 2020). While Type I MOBs are frequently regarded as r-selected species due to their high proliferation rates but poor ability to survive in adverse environments (Ho et al., 2012; Shiau et al., 2020).

### Microbial assemblages in the three maar lakes

The seasonal variability of tropical freshwater environments also influences the diversity of bacterial communities. Results of the PCoA analysis by Bray-Curtis dissimilarity metrics in Lake Yambo showed that bacterial community compositions were different during the transition of the prevailing wind to the NE monsoon (November) than those in September and December. In terms of bacterial species distribution and richness, however, SW stratification (September) showed higher phylogenetic variations than December mixed month (Supplementary Figures 1B, C). Microbial communities are homogeneous under mixing conditions due to the physicochemical characteristics of the water body; however, stratification reveals distinct community patterns between the nutrient-poor, warmer epilimnion, and the nutrient-rich, predominantly anoxic hypolimnion (Lee et al., 2015; Ozbayram et al., 2022).

The Illumina 16S rRNA gene metabarcoding revealed the presence of bacterial and archaeal phyla in the water column. Common freshwater bacterial phyla are also found in the studied maar lakes, where most bacteria in the epilimnion are Actinobacteria, Cyanobacteria, Bacteroidetes, three classes of Proteobacteria, and Verrucomicrobia) while Firmicutes, Bacteroidetes, and Proteobacteria predominate in the hypolimnion (Figures 5, 6) (Newton et al., 2011). The continued monitoring of Lake Yambo in 2022 has shown that the diversity of bacterial communities varies depending on the depth and month (Figure 9). In fact, Shannon diversity indices (H’) measured in 2022 ranged from 6 to 7.5 which means there is a high bacterial diversity in Lake Yambo that is rarely achieved (Ortiz-Burgos, 2016; Bobbitt, 2022). Epilimnion (5 m) depths in both September and December (2022) also have similar diverse bacterial taxa identified (PCoA, Pielou’s evenness) (Figure 9 and Supplementary Figure 1B). The lake’s upper depths were characterized by relatively warm water, where the majority of photosynthesis occurs. The phylum Cyanobacteria was more abundant in the epilimnion depths (5 m) of the three lakes and in most tropical lakes, as their physiological processes require sunlight and oxic DO levels (Madigan et al., 2008). Actinobacteria are also common in any lake of varying trophic status with their abundance inversely correlated to DO concentrations and directly correlated to nutrient availability (Allgaier et al., 2007; Wu et al., 2007; Humbert et al., 2009). The same case in the maar lakes applies wherein Actinobacteria were most abundant in the epilimnion (Figures 5, 6), as these were the depths wherein DO concentrations were adamant and CH_4_ concentrations were relatively lower compared to the deeper layers of the lake (Supplementary Table 1). This is shown by the beta diversity analysis (Supplementary Figure 1D) wherein the bacterial communities in the hypolimnion layer (30–32 m) of Lake Yambo are highly dissimilar to that of the epilimnion (5 m). It is also because the epilimnion is often referred to as the trophogenic zone of lentic systems, where mixing and photosynthesis exceed respiration, whereas the hypolimnion is known as the tropholytic zone, where organic materials are synthesized and mineralized by specific bacterial species (Brusseau et al., 2019). Moreover, the occurrence of the phylum Bacteroidota was seen in nearly all the depths of the lakes, with a more dispersed and higher distribution in Lake Pandin (Figure 5). This bacterial phylum was usually found in sites from other studies wherein dissolved organic carbon and algal-derived carbon input are high (Eiler and Bertilsson, 2004, Kolmonen et al., 2004). Several members of this phylum were found to be active degraders of chitinous and plant-based materials (Tu et al., 2017). Thus, CH_4_ may potentially be higher in the lake’s deepest layer during the SW (April and May) due to sediment productivity. The phylum Proteobacteria, on the other hand, were common members of bacterial communities in oceans and freshwater habitats, mainly because the bacteria in this phylum have different nutrient preferences (i.e., nitrogen and phosphorous) (Newton et al., 2011). The predominance of Alphaproteobacteria (type II) in oligotrophic and acidic humic-rich environments has been reported (Heyer et al., 2002; Hutalle-Schmelzer et al., 2010).

The abundance of the phylum Verrucomicrobia was also seen to be relatively abundant in north- temperate lakes and two shallow lakes with tropical origins (Avila et al., 2017; Chiang et al., 2018). These were also generally found in various depths of the lakes as these bacteria have various metabolic strategies and some members of this phylum are mostly seen in environments with high nutrient content (Haukka et al., 2006; [Bibr B89][Bibr B37]). Like proteobacterial MOB groups, Verrucomicrobial MOBs are believed to play a crucial role in CH_4_-oxidation in extreme environments where they were first discovered (i.e., geothermal and volcanic environments) (Islam et al., 2008; Schmitz et al., 2021).

## Conclusion and future directions

This study showed the presence of three MOB types (I, II, and NC10), and other CH_4_-oxidizing microorganisms from the verrucomicrobia phylum (Methylacidiphilaceae), and archaeal ANME- 1 phylum using several molecular methods in the water column of the three maar lakes.

Community compositions in Lake Yambo were found to be varied with seasonal changes and more distinct during SW stratification through analyses of microbial diversity and distribution. Physicochemical factors such as temperature and pH were significantly correlated with type I MOB, while temperature, DO, and pH were significantly correlated with NC10 during the stratification months of August 2018 and May 2022, respectively. Investigation of the entire microbial diversity of MOBs will require the combination of molecular methodologies such as enrichment cultures, nitrate mineral salts (NMS), CARD-FISH, gene sequencing (16S analysis and pmoA gene analysis), PLFA biomarkers, stable isotope probing, and mRNA-based analyses, as supplemented by various studies from other latitudinal regions. In future studies, CH_4_-oxidation rates (using the ^3^H-CH_4_ tracer technique) by these MOBs and other CH_4_-oxidizers should also be considered to fully understand their roles in mitigating CH_4_ emissions from tropical deep-waters with CH_4_-rich and anoxic water columns. Consequently, CH_4_ production by methanogens and their diversity in tropical lakes should be further investigated using a variety of primer combinations in order to completely understand how CH_4_ dynamics result from the interaction of these species and possibly with other coexisting species. Other physicochemical (i.e., nitrogen and ammonium) and weather parameters should also be considered as drivers of MOB proliferation to gain a more concrete understanding of their roles in CH_4_-oxidation. The very existence of MOBs and the variation in their abundances in the selected Philippine maar lakes show the potential ability of natural ecosystems to minimize the CH_4_ atmospheric emissions, hence aiding in the mitigation of global warming.

## Data availability statement

The datasets presented in this study can be found in online repositories. The names of the repository/repositories and accession number(s) can be found in the article/Supplementary material.

## Ethics statement

This article only studied microbial population and their distribution (without intervention with human or other forms of animals), thus, do not require ethical considerations.

## Author contributions

IB: Data curation, Formal analysis, Investigation, Methodology, Visualization, Writing – original draft. KP: Conceptualization, Data curation, Formal analysis, Investigation, Methodology, Visualization, Writing – original draft, Writing – review and editing. T-HT: Conceptualization, Formal analysis, Methodology, Validation, Writing – review and editing. MM-P: Conceptualization, Investigation, Methodology, Resources, Supervision, Validation, Writing – review and editing. CV: Conceptualization, Formal analysis, Investigation, Methodology, Supervision, Validation, Visualization, Writing – review and editing. JL: Conceptualization, Data curation, Formal analysis, Investigation, Methodology, Software, Validation, Visualization, Writing – review and editing. KP: Writing – original draft. EA: Conceptualization, Investigation, Methodology, Resources, Supervision, Validation, Writing – review and editing. ML: Conceptualization, Formal analysis, Methodology, Software, Validation, Visualization, Writing – original draft, Writing – review and editing. YK: Conceptualization, Methodology, Writing – review and editing. F-KS: Methodology, Investigation, Validation, Writing – review and editing. RP: Investigation, Methodology, Project administration, Resources, Supervision, Validation, Writing – original draft, Writing – review and editing. NO: Data curation, Funding acquisition, Investigation, Methodology, Project administration, Resources, Supervision, Validation, Writing – original draft, Writing – review and editing. P-LW: Data curation, Investigation, Methodology, Writing – original draft. L-HL: Data curation, Formal analysis, Funding acquisition, Investigation, Methodology, Project administration, Resources, Supervision, Validation, Writing – original draft, Writing – review and editing. WC: Data curation, Methodology, Investigation, Validation, Writing – original draft.

## References

[B1] Aguilar J. I. Mendoza-Pascual M. U. Padilla K. S. A. R. Papa R. D. S. Okuda N. (2023). Mixing regimes in a cluster of seven maar lakes in tropical Monsoon Asia. *Inland Waters* 13 47–61. 10.1080/20442041.2023.2167484

[B2] Allgaier M. Brückner S. Jaspers E. Grossart H. P. (2007). Intra- and inter-lake variability of free-living and particle-associated Actinobacteria communities. *Environ. Microbiol.* 9 2728–2741. 10.1111/j.1462-2920.2007.01385.x 17922757

[B3] Amann R. Fuchs B. (2008). Single-cell identification in microbial communities by improved fluorescence in-situ hybridization techniques. *Nat. Rev. Microbiol.* 6 339–348. 18414500 10.1038/nrmicro1888

[B4] Anderson M. J. (2001). A new method for non-parametric multivariate analysis of variance. *Austral Ecol.* 26 32–46. 10.1111/j.1442-9993.2001.01070.pp.x

[B5] Avila M. P. Staehr P. A. Barbosa F. A. R. Chartone-Sauza E. Nascimento A. M. A. (2017). Seasonality of freshwater bacterioplankton diversity in two tropical shallow lakes from the Brazilian Atlantic Forest. *FEMS Microbiol. Ecol.* 93. 15–28. 10.1093/femsec/fiw218 27797965

[B6] Bannister W. McGowan S. Santos-Borja A. C. Quak J. Fong L. S. Mendoza M. (2019). Potential anthropogenic regime shifts in three freshwater lakes in tropical East Asia. *Freshw. Biol.* 64 708–722. 10.1111/fwb.13256

[B7] Bastviken D. Cole J. (2008). Fates of methane from different lake habitats: Connecting whole-lake budgets and CH_4_ emissions. *J. Geophys. Res*. 113:G02024. 10.1029/2007JG000608

[B8] Bastviken D. Cole J. Pace M. Tranvik L. (2004). Methane emissions from lakes: Dependence of lake characteristics, two regional assessments, and a global estimate. *Glob. Biogeochem. Cycles* 18:GB4009. 10.1029/2004GB002238

[B9] Bastviken D. Ejlertsson J. Tranvik L. (2002). Measurement of methane oxidation in lakes: a comparison of methods. *Environ. Sci. Technol.* 36 3354–3361. 10.1021/es010311p 12188365

[B10] Bastviken D. Ejlertsson J. Sundh I. Tranvik L. (2003). Methane as a source of carbon and energy for lake pelagic food webs. *Ecology* 84 969–981.

[B11] Bastviken D. Tranvik L. J. Downing J. A. Crill P. M. Enrich-Prast A. (2011). Freshwater methane emissions offset the continental carbon sink. *Science* 331:50. 10.1126/science.1196808 21212349

[B12] Beaulieu J. J. DelSontro T. Downing J. A. (2019). Eutrophication will increase methane emissions from lakes and impoundments during the 21st century. *Nat. Commun. Nat.* 10:1375. 10.1038/s41467-019-09100-5 30914638 PMC6435651

[B13] Blees J. Niemann H. Wenk C. Zopfi J. Schubert C. Kirf M. (2014). Micro-aerobic methane oxidation in the chemocline and anoxic water column of deep south-Alpine Lake Lugano (Switzerland). *Limnol. Oceanogr.* 59 311–324.

[B14] Blumenberg M. Seifert R. Reitner J. Pape T. Michaelis W. (2004). Membrane lipid patterns typify distinct anaerobic methanotrophic consortia. *Proc. Natl. Acad. Sci. U S A.* 101 11111–11116. 10.1073/pnas.0401188101 15258285 PMC503748

[B15] Bobbitt Z. (2022). *Shannon diversity index: Definition & example*. Statology. Available online at: https://www.statology.org/shannon-diversity-index/ (accessed June 12, 2023).

[B16] Bodrossy L. Holmes E. M. Holmes A. J. Kovacs K. L. Murrell J. C. (1997). Analysis of 16S rRNA and methane monooxygenase gene sequence reveals a novel group of thermotolerant and thermophilic methanotrophs. *Methylocaldum gen. Nov. Arch. Microbiol.* 168 493–503. 10.1007/s002030050527 9385141

[B17] Boetius A. Ravenschlag K. Schubert C. Rickert D. Widdel F. Gieseke A. (2000). A marine microbial consortium apparently mediating anaerobic oxidation of methane. *Nature* 407 623–626. 11034209 10.1038/35036572

[B18] Borjesson G. Sundh I. Svensson B. (2004). Microbial oxidation of CH_4_ at different temperatures in landfill cover soils. *FEMS Microbiol. Ecol.* 48 305–312. 19712300 10.1016/j.femsec.2004.02.006

[B19] Borrel G. Jezequel D. Biderre-Petit C. Morel-Desrosiers N. Morel J. Peyret P. (2011). Production and consumption of methane in freshwater lake ecosystems. *Res. Microbiol.* 162 832–847. 21704700 10.1016/j.resmic.2011.06.004

[B20] Bourne D. G. McDonald I. R. Murrell J. C. (2001). Comparison of pmoA PCR primer sets as tools for investigating methanotroph diversity in three Danish soils. *Appl. Environ. Microbiol.* 67 3802–3809. 10.1128/AEM.67.9.3802-3809.2001 11525970 PMC93094

[B21] Bowman J. P. (2014). “The family methylococcaceae,” in *The Prokaryotes – Gammaproteobacteria*, ed. Rosenber E. (Berlin: Springer).

[B22] Bray J. R. Curtis J. T. (1957). An ordination of the upland forest communities of Southern Wisconsin. *Ecol. Monogr.* 27 325–349. 10.2307/1942268

[B23] Briddon C. L. Metcalfe S. Taylor D. Bannister W. Cunanan M. Santos-Borja A. C. (2022). Changing water quality and thermocline depth along an aquaculture gradient in six tropical crater lakes. *Hydrobiologia* 850 283–299. 10.1007/s10750-022-05065-7

[B24] Brillo B. B. C. (2016a). Development issues of a small transboundary lake: Yambo Lake of San Pablo City, Nagcarlan and Rizal, Laguna, Philippines. *Soc. Sci.* 11 5693–5702.

[B25] Brillo B. B. C. (2016b). An assessment of development of a transboundary small lake: Calibato Lake, San Pablo City and Rizal, Laguna, The Philippines. *Asian J. Water, Environ. Pollut.* 13 55–67.

[B26] Brusseau M. Walker D. Fitzsimmons K. (2019). *Physical-Chemical Characteristics of Water.* Amsterdam: Elsevier eBooks.

[B27] Callahan B. J. McMurdie P. J. Rosen M. J. Han A. W. Johnson A. J. A. Holmes S. P. (2016). DADA2: High-resolution sample inference from Illumina amplicon data. *Nat. Methods* 13 581–583. 10.1038/nmeth.3869 27214047 PMC4927377

[B28] Caporaso J. G. Lauber C. L. Walters W. A. Berg-Lyons D. Lozupone C. A. Turnbaugh P. J. (2011). Global patterns of 16S rRNA diversity at a depth of millions of sequences per sample. *Proc. Natl. Acad. Sci.* 108 4516–4522. 10.1073/pnas.1000080107 20534432 PMC3063599

[B29] Carini S. Bano N. LeCleir G. Joye S. B. (2005). Aerobic methane oxidation and methanotroph community composition during seasonal stratification in Mono Lake, California (USA). *Environ. Microbiol.* 7 1127–1138. 10.1111/j.1462-2920.2005.00786.x 16011750

[B30] Chang C.-Y. Tung H.-H. Tseng I.-C. Wu J.-H. Liu Y.-F. Lin H.-M. (2010). Dynamics of methanotrophic communities in tropical alkaline landfill upland soil. *Appl. Soil Ecol.* 46 192–199. 10.1016/j.apsoil.2010.08.009

[B31] Chao A. (1984). Nonparametric estimation of the number of classes in a population. *Scand. J. Stat.* 11 265–270.

[B32] Chiang E. Schmidt M. L. Berry M. A. Biddanda B. A. Burtner A. Johengen T. H. (2018). Verrucomicrobia are prevalent in north-temperate freshwater lakes and display class-level preferences between lake habitats. *PLoS One* 13:e0195112. 10.1371/journal.pone.0195112 29590198 PMC5874073

[B33] Costello A. M. Lidstrom M. E. (1999). Molecular characterization of functional and phylogenetic genes from natural populations of methanotrophs in lake sediments. *Appl. Environ. Microbiol.* 65 5066–5074. 10543824 10.1128/aem.65.11.5066-5074.1999 PMC91682

[B34] Costello A. M. Auman A. J. Macalady J. L. Scow K. M. Lidstrom M. E. (2002). Estimation of methanotroph abundance in a freshwater lake sediment. *Environ. Microbiol.* 4 443–450. 10.1046/j.1462-2920.2002.00318.x 12153585

[B35] Crevecoeur S. Vincent W. F. Comte J. Matveev A. Lovejoy C. (2017). Diversity and potential activity of methanotrophs in high-methane emitting permafrost thaw ponds. *PLoS One* 12:e0188223. 10.1371/journal.pone.0188223 29182670 PMC5705078

[B36] De Leon J. To, Peralta E. M. Salluta J. C. R. (2024). Identifying the mixing regime of Lake Taal, Batangas, Philippines: Implications of lake mixing and stratification to lake management article history. *NRCP Res. J.* 23 26–39.

[B37] De Wever A. Van der Gucht K. Muylaert K. Cousin S. Vyverman W. (2008). Clone library analysis reveals an unusual composition and strong habitat partitioning of pelagic bacterial communities in Lake Tanganyika. *Aquatic Microbial Ecol.* 50 113–122.

[B38] Dedysh S. N. Beletsky A. V. Ivanova A. A. Danilova O. V. Begmatov S. Kulichevskaya I. S. (2021). Peat-inhabiting verrucomicrobia of the order methylacidiphilales do not possess methanotrophic capabilities. *Microorganisms* 9:2566. 10.3390/microorganisms9122566 34946166 PMC8706344

[B39] Dedysh S. Liesack W. Khmelenina V. Suzina N. Trotsenko Y. Semrau J. (2000). *Methylocella palustris* gen. nov., sp. nov., a new methane-oxidizing acidophilic bacterium from peat bogs, representing a novel subtype of serine- pathway methanotrophs. *Int. J. Syst. Evol. Microbiol.* 50 955–969. 10.1099/00207713-50-3-955 10843033

[B40] Deines P. Fink P. (2011). The potential of methanotrophic bacteria to compensate for food quantity or food quality limitations on Daphnia. *Aquatic Microb. Ecol.* 65 197–206.

[B41] DelSontro T. Beaulieu J. J. Downing J. A. (2018). Greenhouse gas emissions from lakes and impoundments: upscaling in the face of global change. *Limnol. Oceanogr. Lett.* 3 64–75. 10.1002/lol2.10073 32076654 PMC7029703

[B42] Deutzmann J. S. Schink B. (2011). Anaerobic oxidation of methane in sediments of lake constance, an oligotrophic freshwater lake. *Appl. Environ. Microbiol.* 77 4429–4436. 10.1128/AEM.00340-11 21551281 PMC3127698

[B43] Dumestre J. F. Casamayor E. O. Massana R. Pedros-Alio C. (2002). Changes in bacterial and archaeal assemblages in an equatorial river induced by the water eutrophication of Petit Saut dam reservoir (French Guiana). *Aquatic Microb. Ecol.* 26 209–221.

[B44] Dumont M. Murrell J. C. (2005). Community-level analysis: key genes of aerobic methane oxidation. *Methods Enzymol.* 397 413–427. 16260306 10.1016/S0076-6879(05)97025-0

[B45] Dunfield P. F. Yuryev A. Senin P. Smirnova A. V. Stott M. B. Hou S. (2007). Methane oxidation by an extremely acidophilic bacterium of the phylum Verrucomicrobia. *Nature* 450, 879–882. 10.1038/nature06411 18004300

[B46] Durisch-Kaiser E. Schmid M. Peeters F. Kipfer R. Dinkel C. Diem T. (2011). What prevents outgassing of methane to the atmosphere in Lake Tanganyika. *J. Geophys. Res*. 116. 10.1029/2010JG001323

[B47] Edgar R. C. Haas B. J. Clemente J. C. Quince C. Knight R. (2011). UCHIME improves sensitivity and speed of chimera detection. *Bioinformatics* 27 2194–2200. 10.1093/bioinformatics/btr381 21700674 PMC3150044

[B48] Eiler A. Bertilsson S. (2004). Composition of freshwater bacterial communities associated with cyanobacterial blooms in four Swedish lakes. *Environ. Microbiol.* 6 1228–1243. 10.1111/j.1462-2920.2004.00657.x 15560821

[B49] Eller G. Kanel L. Kruger M. (2005). Cooccurrence of aerobic and anaerobic methane oxidation in the water column of lake plußsee. *Appl. Environ. Microbiol.* 71 8925–8928. 10.1128/AEM.71.12.8925-8928.2005 16332891 PMC1317442

[B50] Eller G. Stubner S. Frenzel P. (2001). Group-specific 16S rRNA targeted probes for the detection of type I and type II methanotrophs by fluorescence in situ hybridisation. *FEMS Microbiol. Lett.* 198 91–97. 11430414 10.1111/j.1574-6968.2001.tb10624.x

[B51] Esposito C. Nijman T. P. Veraart A. J. Audet J. Levi E. E. Lauridsen T. L. (2023). Activity and abundance of methane-oxidizing bacteria on plants in experimental lakes subjected to different nutrient and warming treatments. *Aquatic Bot.* 185:103610. 10.1016/j.aquabot.2022.103610

[B52] Estaki M. Jiang L. Bokulich N. A. McDonald D. González A. Kosciolek T. (2020). QIIME 2 enables comprehensive end-to-end analysis of diverse microbiome data and comparative studies with publicly available data. *Curr. Protoc. Bioinformatics* 70:e100. 10.1002/cpbi.100 32343490 PMC9285460

[B53] Ettwig K. van Allen T. van de Pas-Schoonen K. Jetten M. Strous M. (2009). Enrichment and mechanical detection of denitrifying methanotrophic bacteria of the NC10 phylum. *Appl. Environ. Microbiol.* 75 3656–3662. 10.1128/AEM.00067-09 19329658 PMC2687271

[B54] Faith D. P. (1992). Conservation evaluation and phylogenetic diversity. *Biol. Conserv.* 61 1–10. 10.1016/0006-3207(92)91201-3

[B55] Forster P. Ramaswamy V. Artaxo P. Berntsen T. Betts R. Fahey D. W. (2007). “Changes in atmospheric constituents and in radiative forcing,” in *Climate Change 2007: the Physical Science Basis. Contribution of Working Group I to the Fourth Assessment Report of the Intergovernmental Panel on Climate Change*, eds Solomon S. Qin M. Manning Z. Chen M. Marquis K. B. Averyt M. (Cambridge: Cambridge University Press).

[B56] Gagliano A. L. D’Alessandro W. Tagliavia M. Parello F. Quatrini P. (2014). Methanotrophic activity and diversity of methanotrophs in volcanic geothermal soils at Pantelleria (Italy). *Biogeosciences* 11 5865–5875. 10.5194/bg-11-5865-2014

[B57] Graef C. Hestnes A. G. Svenning M. M. Frenzel P. (2011). The active methanotrophic community in a wetland from the High Arctic. *Environ. Microbiol. Rep.* 3 466–472. 10.1111/j.1758-2229.2010.00237.x 23761309

[B58] Graf J. S. Mayr M. J. Marchant H. K. Tienken D. Hach P. F. Brand A. (2018). Bloom of a denitrifying methanotroph, ‘Candidatus *Methylomirabilis limnetica*’, in a deep stratified lake. *Environ. Microbiol.* 20 2598–2614. 10.1111/1462-2920.14285 29806730

[B59] Green P. N. Ardley J. K. (2018). Review of the genus Methylobacterium and closely related organisms: a proposal that some Methylobacterium species be reclassified into a new genus, Methylorubrum gen. nov. *Int. J. Syst. Evol. Microbiol.* 68 2727–2748. 10.1099/ijsem.0.002856 30024371

[B60] Guerrero-Cruz S. Vaksmaa A. Horn M. A. Niemann H. Pijuan M. Ho A. (2021). Methanotrophs: discoveries, environmental relevance, and a perspective on current and future applications. *Front. Microbiol.* 12:678057. 10.3389/fmicb.2021.678057 34054786 PMC8163242

[B61] Guggenheim C. Freimann R. Mayr M. J. Beck K. Wehrli B. Bürgmann H. (2020). Environmental and microbial interactions shape methane-oxidizing bacterial communities in a stratified lake. *Front. Microbiol.* 11:579427. 10.3389/fmicb.2020.579427 33178162 PMC7593551

[B62] Gulledge J. Ahmad A. Steudler P. A. Pomerantz W. J. Cavanaugh C. M. (2001). Family- and genus-level 16S rRNA-targeted oligonucleotide probes for ecological studies of methanotrophic bacteria. *Appl. Environ. Microbiol.* 67 4726–4733. 10.1128/AEM.67.10.4726-4733.2001 11571178 PMC93225

[B63] Haukka K. Kolmonen E. Hyder R. Hietala J. Vakkilainen K. Kairesalo T. (2006). Effect of nutrient loading on bacterioplankton community composition in lake mesocosms. *Microbial Ecology* 51 137–146. 10.1007/s00248-005-0049-7 16435168

[B64] He R. Su Y. Leewis M. C. Chu Y. X. Wang J. Ma R. C. (2020). Low O2 level enhances CH_4_-derived carbon flow into microbial communities in landfill cover soils. *Environ. Pollut.* 258:113676. 10.1016/j.envpol.2019.113676 31818614

[B65] He Z. Cai C. Wang J. Xu X. Zheng P. Jetten M. S. (2016). A novel denitrifying methanotroph of the NC10 phylum and its microcolony. *Sci. Rep.* 6:32241. 10.1038/srep32241 27582299 PMC5007514

[B66] Heyer J. Galchenko V. F. Dunfield P. F. (2002). Molecular phylogeny of type II methane-oxidizing bacteria isolated from various environments. *Microbiology* 148 2831–2846. 12213929 10.1099/00221287-148-9-2831

[B67] Ho A. Kerckhof F. M. Luke C. Reim A. Krause S. Boon N. (2012). Conceptualizing functional traits and ecological characteristics of methane-oxidizing bacteria as life strategies. *Environ. Microbiol. Rep.* 5 335–345. 10.1111/j.1758-2229.2012.00370.x 23754714

[B68] Holmes A. J. Tujula N. A. Holley M. Contos A. James J. M. Rogers P. (2001). Phylogenetic structure of unusual aquatic microbial formations in Nullarbor caves. *Australia. Environ. Microbiol.* 3 256–264. 10.1046/j.1462-2920.2001.00187.x 11359511

[B69] Humbert J. F. Dorigo U. Cecchi P. Le Berre B. Debroas D. Bouvy M. (2009). Comparison of the structure and composition of bacterial communities from temperate and tropical freshwater ecosystems. *Environ. Microbiol.* 11 2339–2350. 19508336 10.1111/j.1462-2920.2009.01960.x

[B70] Huse S. M. Welch D. M. Morrison H. G. Sogin M. L. (2010). Ironing out the wrinkles in the rare biosphere through improved OTU clustering. *Environ. Microbiol.* 12 1889–1898. 10.1111/j.1462-2920.2010.02193.x 20236171 PMC2909393

[B71] Hutalle-Schmelzer K. M. L. Zwirnmann E. Krüger A. Grossart H. (2010). Enrichment and cultivation of pelagic bacteria from a humic lake using phenol and humic matter additions. *FEMS Microb. Ecol.* 72 58–73. 10.1111/j.1574-6941.2009.00831.x 20459514

[B72] Iguchi A. Iyoda S. Kikuchi T. Ogura Y. Katsura K. Ohnishi M. (2014). A complete view of the genetic diversity of the *Escherichia coli* O-antigen biosynthesis gene cluster. *DNA Res.* 22 101–107. 10.1093/dnares/dsu043 25428893 PMC4379981

[B73] Islam T. Jensen S. Reigstad L. J. Larsen Ø Birkeland N. K. (2008). Methane oxidation at 55^°^C and pH 2 by a thermoacidophilic bacterium belonging to the Verrucomicrobia phylum. *Proc. Natl. Acad. Sci. U.S.A.* 105 300–304. 10.1073/pnas.0704162105 18172218 PMC2224206

[B74] Itoh M. Kobayashi Y. Chen T. Tokida T. Fukui M. Kojima H. (2015). Effect of interannual variation in winter vertical mixing on CH_4_ dynamics in a subtropical reservoir. *J. Geophys. Res. Biogeosci.* 120 1246–1261.

[B75] Johnson M. S. Matthews E. Du J. Genovese V. Bastviken D. (2022). Methane emission from global lakes: new spatiotemporal data and observation-driven modeling of methane dynamics indicates lower emissions. *JGR Biogeosci.* 127:e2022JG006793. 10.1029/2022JG006793 36250198 PMC9540782

[B76] Juutinen S. Rantakari M. Kortelainen P. Huttunen J. T. Larmola T. Alm J. (2009). Methane dynamics in different boreal lake types. *Biogeosciences* 6 209–223. 10.5194/bg-6-209-2009

[B77] Kalyuzhnaya M. G. Gomez O. A. Murrell J. C. (2019). “The methane-oxidizing bacteria (Methanotrophs),” in *Taxonomy, Genomics and Ecophysiology of Hydrocarbon-Degrading Microbes*, ed. McGenity T. J. (Berlin: Springer).

[B78] Kaupper T. Mendes L. W. Lee H. J. Mo Y. Poehlein A. Jia Z. (2021). When the going gets tough: emergence of a complex methane-driven interaction network during recovery from desiccation-rewetting. *Soil Biol. Biochem.* 153:108109. 10.1016/j.soilbio.2020.108109

[B79] Kirschke S. Bousquet P. Ciais P. Saunois M. Canadell J. G. Dlugokencky E. J. (2013). The decades of global methane sources and sinks. *Nat. Geosci.* 6 813–231.

[B80] Knittel K. Lösekann T. Boetius A. Kort R. Amann R. (2005). Diversity and distribution of methanotrophic archaea at cold seeps. *Appl. Environ. Microbiol.* 71 467–479. 15640223 10.1128/AEM.71.1.467-479.2005 PMC544223

[B81] Kobayashi Y. Kojima H. Itoh M. Okuda N. Fukui M. Shiah F. (2016). Abundance of planktonic methane-oxidizing bacteria in a subtropical reservoir. *Plankton Benthos Res.* 11 144–146. 10.1038/srep05728 PMC4124587 25098653

[B82] Kojima H. Iwata T. Fukui M. (2009). DNA-based analysis of planktonic methanotrophs in a stratified lake. *Freshw. Biol.* 54 1501–1509.

[B83] Kojima H. Tokizawa R. Kogure H. Kobayashi Y. Itoh M. Shiah F. (2014). Community structure of planktonic methane-oxidizing bacteria in a subtropical reservoir characterized by dominance of phylotype closely related to nitrite reducer. *Nat. Sci. Rep.* 4:5728. 10.1038/srep05728 25098653 PMC4124587

[B84] Kojima H. Tsutsumi M. Ishikawa K. Iwata T. Mubmann M. Fukui M. (2012). Distribution of putative denitrifying methane oxidizing bacteria in a sediment of a freshwater lake, Lake Biwa. *Syst. Appl. Microbiol.* 35 233–238. 10.1016/j.syapm.2012.03.005 22504019

[B85] Kolmonen E. Sivonen K. Rapala J. Haukka K. (2004). Diversity of cyanobacteria and heterotrophic bacteria in cyanobacterial blooms in Lake Joutikas, Finland. *Aquatic Microbiol. Ecol.* 36 201–211. 10.1038/ismej.2008.110 19020559

[B86] Kubota K. (2013). CARD-FISH for environmental microorganisms: technical advancement and future applications. *Microbes Environ.* 28 3–12. 10.1264/jsme2.me12107 23124765 PMC4070690

[B87] Lambrecht N. Katsev S. Wittkop C. Hall S. J. Sheik C. S. Picard A. (2019). Biogeochemical and physical controls on methane fluxes from two ferruginous meromictic lakes. *Geobiol.* 18 54–69. 10.1111/gbi.12365 31592570

[B88] Lee H. J. Jeong S. E. Kim P. J. Madsen E. L. Jeon C. O. (2015). High resolution depth distribution of Bacteria, Archaea, methanotrophs, and methanogens in the bulk and rhizosphere soils of a flooded rice paddy. *Front. Microbiol.* 6:639. 10.3389/fmicb.2015.00639 26161079 PMC4479796

[B89] Liesack W. Feng L. Wang L. Alam M. (2007). Methane oxidation by an extremely acidophilic bacterium of the phylum Verrucomicrobia. *Nat. Lett.* 450 879–882. 10.1038/nature06411 18004300

[B90] Lin Y. Tu T. Wei C. Rumble D. Lin L. Wang P. (2018). Steep redox gradient and biogeochemical cycling driven by deeply sourced fluids and gases in a terrestrial mud volcano. *FEMS Microbiol. Ecol.* 94:fiy171. 10.1093/femsec/fiy171 30165492

[B91] Liu J. Diao Z. Xu X. Xie Q. (2019). Effects of dissolved oxygen, salinity, nitrogen and phosphorus on the release of heavy metals from coastal sediments. *Sci. Total Environ.* 666 894–901. 10.1016/j.scitotenv.2019.02.288 30818213

[B92] LLDA (2014). *Laguna Lake Development Authority. Annual Report 2014.* Quezon: LLDA.

[B93] Luesken F. A. Wu M. L. Op den Camp H. J. M. Keltjens J. T. Stunnenberg H. Francoijs K.-J. (2012). Effect of oxygen on the aerobic methanotroph ‘Candidatus Methylomirabilis oxyfera’: kinetic and transcriptional analysis. *Environ. Microbiol.* 14 1024–1034. 10.1111/j.1462-2920.2011.02682.x 22221911

[B94] Luke C. Frenzel P. Ho A. Flantis D. Schad P. Schneider B. (2014). Macroecology of methane-oxidizing bacteria: the β diversity of pmoA genotypes in tropical and subtropical rice paddies. *Environ. Microbiol.* 16 72–83. 24914433 10.1111/1462-2920.12190

[B95] Madigan M. T. Martinko J. M. Dunlap P. V. Clark D. P. (2008). *Brock Biology of Microorganisms*, 12th Edn. Upper Saddle River, NJ: Prentice-Hall.

[B96] Manjarrés D. Montemurro N. Pérez S. (2022). Development of a USE/d-SPE and targeted DIA-Orbitrap-MS acquisition methodology for the analysis of wastewater-derived organic pollutants in fish tissues and body fluids. *MethodsX* 9:101705. 10.1016/j.mex.2022.101705 35518922 PMC9062737

[B97] Marinho C. C. Palma Silva C. Albertoni E. F. Trindade C. R. Esteves F. A. (2009). Seasonal dynamics of methane in the water column of two subtropical lakes differing in trophic status. *Braz. J. Biol.* 69 281–287. 10.1590/s1519-69842009000200007 19675928

[B98] Martzy R. Bica-Schröder K. Pálvölgyi D. M. Kolm C. Jakwerth S. Kirschner A. K. (2019). Simple lysis of bacterial cells for DNA-based diagnostics using hydrophilic ionic liquids. *Sci. Rep.* 9:13994. 10.1038/s41598-019-50246-5 31570727 PMC6768989

[B99] Mayr M. J. Zimmermann M. Dey J. Brand A. Wehrli B. Burgmann H. (2020). Growth and rapid succession of methanotrophs effectively limit methane release during lake overturn. *Commun. Biol.* 3:108. 10.1038/s42003-020-0838-z 32144394 PMC7060174

[B100] Mendoza M. U. Briones J. C. A. Itoh M. Padilla K. S. A. R. Aguilar J. I. Okuda N. (2019). Small Maar lakes of Luzon Island, Philippines: their limnological status and implications on the management of tropical lakes – a review. *Philippine J. Sci.* 148 565–578.

[B101] Mendoza-Pascual M. U. Itoh M. Aguilar J. Padilla K. S. A. R. Papa R. D. S. Okuda N. (2021). Controlling factors of methane in tropical lakes of different depths. *J. Geophys. Res. Biogeosci.* 126:e2020JG005828. 10.1029/2020jg005828

[B102] Merino-Ibarra M. Ramírez-Zierold J. A. Valdespino-Castillo P. M. Castillo-Sandoval F. S. Guzmán-Arias A. P. Barjau-Aguilar M. (2021). Vertical boundary mixing events during stratification govern heat and nutrient dynamics in a windy tropical reservoir lake with important water-level fluctuations: a long-term (2001– 2021) study. *Water* 13:3011. 10.3390/w13213011

[B103] Michaelis W. Seifert R. Nauhaus K. Truede T. Thiel V. Blumenberg M. (2002). Microbial reefs in the black sea fueled by anaerobic oxidation of methane. *Science* 297 1013–1015. 10.1126/science.1072502 12169733

[B104] Milucka J. Kirf M. Lu L. Krupke A. Lam P. Littmann S. (2015). Methane oxidation coupled to oxygenic photosynthesis in anoxic waters. *ISME J.* 9 1991–2002. 25679533 10.1038/ismej.2015.12 PMC4542029

[B105] Ming T. Li W. Yuan Q. Davies P. de Richter R. Peng C. (2022). Perspectives on removal of atmospheric methane. *Adv. Appl. Energy* 5:100085. 10.1016/j.adapen.2022.100085

[B106] Mohammadi S. S. Schmitz R. A. Pol A. Berben T. Jetten M. S. M. Op (2019). The acidophilic methanotroph Methylacidimicrobium tartarophylax 4AC grows as autotroph on H2 under microoxic conditions. *Front. Microbiol.* 10:2352. 10.3389/fmicb.2019.02352 31681216 PMC6813726

[B107] Naqvi S. W. A. Lam P. Narvenkar G. Sarkar A. Naik H. Pratihary A. (2018). Methane stimulates massive nitrogen loss from freshwater reservoirs in India. *Nat. Commun.* 9:1265. 10.1038/s41467-018-03607-z 29593290 PMC5871758

[B108] Natarajan V. P. Zhang X. Morono Y. Inagaki F. Wang F. (2016). A modified SDS-based DNA extraction method for high quality environmental DNA from seafloor environments. *Front. Microbiol.* 7:986. 10.3389/fmicb.2016.00986 27446026 PMC4917542

[B109] Navarrete I. A. Dicen G. P. Perez T. R. Mendoza S. M. Rallos R. V. Labides J. L. R. (2019). Towards integrated management of a shallow tropical lake: assessment of water quality, sediment geochemistry, and phytoplankton diversity in Lake Palakpakin. *Philippines. Environ. Monitoring Assess.* 191:485. 10.1007/s10661-019-7617-7 31280379

[B110] Newton R. J. Jones S. E. Eiler A. McMahon K. D. Bertilsson S. (2011). A guide to the natural history of freshwater lake bacteria. *Microbiol. Mol. Biol. Rev.* 75 14–49. 10.1128/MMBR.00028-10 21372319 PMC3063352

[B111] Orphan V. J. Hinrichs K. U. Ussler W. Paull C. K. Taylor L. T. Sylva S. P. (2001). Comparative analysis of methane-oxidizing archaea and sulfate-reducing bacteria in anoxic marine sediments. *Appl. Environ. Microbiol.* 67 1922–1934. 10.1128/aem.67.4.1922-1934.2001 11282650 PMC92814

[B112] Ortiz-Burgos S. (2016). “Shannon-weaver diversity index,” in *Encyclopedia of Estuaries. Encyclopedia of Earth Sciences Series*, ed. Kennish M. J. (Dordrecht: Springer).

[B113] Oswald K. Milucka J. Brand A. Littmann S. Wehrli B. Kuypers M. M. M. (2015). Light-dependent aerobic methane oxidation reduces methane emissions from seasonally stratified lakes. *PLoS One* 10:e0132574. 10.1371/journal.pone.0132574 26193458 PMC4508055

[B114] Ozbayram E. G. Köker L. Çam A. O. Akçaalan R. Albay M. (2022). Temporal and spatial variations of the bacterial diversity in a deep Alkaline Lake. *Water* 14:4097. 10.3390/w14244097

[B115] Pasche N. Schmid M. Vazquez F. Schubert C. J. Wuest A. Kessler J. D. (2011). Methane sources and sinks in Lake Kivu. *J. Geophys. Res*. 116:G03006. 10.1029/2011JG001690

[B116] Pavlekovic M. Schmid M. C. Schmider-Poignee N. Spring S. Pilhofer M. Gaul T. (2009). Optimization of three FISH procedures for in situ detection of anaerobic ammonium oxidizing bacteria in biological wastewater treatment. *J. Microbiol. Methods* 78 119–126. 10.1016/j.mimet.2009.04.003 19389431

[B117] Pernthaler A. Pernthaler J. Amann R. (2002). Fluorescence in-situ hybridization and catalyzed reporter deposition for the identification of marine bacteria. *Appl. Environ. Microbiol.* 68 3094–3101. 12039771 10.1128/AEM.68.6.3094-3101.2002 PMC123953

[B118] Pol A. Heijmans K. Harhangi H. R. Tedesco D. Jetten M. S. M. Op den Camp H. J. M (2007). Methanotrophy below pH 1 by a new Verrucomicrobia species. *Nat. Lett.* 450 918–921. 10.1038/nature06222 18004305

[B119] Raghoebarsing A. A. Pol A. van de Pas-Schoonen K. T. Smolders A. J. P. Ettwig K. F. Rijpstra I. C. (2006). A microbial consortium couples anaerobic methane oxidation to denitrification. *Nat. Lett.* 440 918–921. 10.1038/nature04617 16612380

[B120] Rappé M. S. Giovannoni S. J. (2003). The uncultured microbial majority. *Annu. Rev Microbiol.* 57 369–394. 14527284 10.1146/annurev.micro.57.030502.090759

[B121] Roland F. A. E. Morana C. Darchambeau F. Cowe S. A. Thamdrup B. Descy J. (2018). Anaerobic methane oxidation and aerobic methane production in an east African great lake (Lake Kivu). *J. Great Lakes Res.* 44 1183–1193. 10.1016/j.jglr.2018.04.003

[B122] Roldán D. M. Carrizo D. Sánchez-García L. Menes R. J. (2022). Diversity and effect of increasing temperature on the activity of methanotrophs in sediments of fildes Peninsula Freshwater Lakes, King George Island, Antarctica. *Front. Microbiol.* 13:822552. 10.3389/fmicb.2022.822552 35369426 PMC8969513

[B123] Rybak M. Cerbin S. Pelechata A. Bartosiewicz M. Bodelier P. (2018). “The influence of eutrophication on methane production and its potential as a carbon source for zooplankton,” in *Proceedings of the 17th World Lake Conference*, (Ibaraki).

[B124] Saarela T. Rissanen A. J. Ojala A. Pumpanen J. Aalto S. Tiirola M. (2020). CH_4_ oxidation in a boreal lake during the development of hypolimnetic hypoxia. *Aquatic Sci.* 82:19. 10.1007/s00027-019-0690-8 32362734 PMC7181431

[B125] Schloss P. D. Westcott S. L. (2011). Assessing and improving methods used in operational taxonomic-unit based approaches for 16S rRNA gene sequence analysis. *Appl. Environ. Microbiol.* 77 3219–3226. 10.1128/AEM.02810-10 21421784 PMC3126452

[B126] Schloss P. D. Westcott S. L. Ryabin T. Hall J. R. Hartmann M. Hollister E. B. (2009). Introducing mothur: open-source, platform-independent, community-supported software for describing and comparing microbial communities. *Appl. Environ. Microbiol.* 75 7537–7541. 10.1128/AEM.01541-09 19801464 PMC2786419

[B127] Schmitz R. A. Peeters S. H. Versantvoort W. Picone N. Pol A. Jetten M. S. M. (2021). Verrucomicrobial methanotrophs: ecophysiology of metabolically versatile acidophiles. *FEMS Microbiol. Rev.* 45:fuab007. 10.1093/femsre/fuab007 33524112 PMC8498564

[B128] Schubert C. J. Diem T. Eugster W. (2012). Methane emissions from a small wind shielded lake determined by eddy covariance, flux chambers, anchored funnels, and boundary model calculations: a comparison. *Environ. Sci. Technol.* 46 4515–4522. 10.1021/es203465x 22436104

[B129] Schubert C. J. Lucas F. S. Durisch-Kaiser E. Stierli R. Diem T. Scheidegger O. (2010). Oxidation and emission of methane in a monomictic lake (Rotsee, Switzerland). *Aquatic Sci.* 72 455–456. 10.1007/s00027-010-0148-5

[B130] Shen L. D. He Z. F. Zhu Q. Chen D. Q. Lou L. P. Xu X. Y. (2012). Microbiology, ecology, and application of the nitrite-dependent anaerobic methane oxidation process. *Front. Microbiol.* 3:269. 10.3389/fmicb.2012.00269 22905032 PMC3408237

[B131] Shiau Y. J. Lin C. W. Cai Y. Jia Z. Lin Y. T. Chiu C. Y. (2020). Niche differentiation of active methane-oxidizing bacteria in estuarine mangrove forest soils in Taiwan. *Microorganisms* 8:1248. 10.3390/microorganisms8081248 32824517 PMC7466156

[B132] Singh J. S. (2013). Anticipated effect of climate change on methanotrophic methane oxidation. *Climate Change Environ. Sustainability* 1 20–24.

[B133] Smith G. J. Wrighton K. C. (2019). Metagenomic approaches unearth methanotroph phylogenetic and metabolic diversity. *Curr. Issues Mol. Biol.* 33 57–84. 10.21775/cimb.033.057 31166185

[B134] Sundh I. Bastviken D. Tranvik L. J. (2005). Abundance, activity, and community structure of pelagic methane-oxidizing bacteria in temperate lakes. *Appl. Environ. Microbiol.* 71 6746–6752. 10.1128/AEM.71.11.6746-6752.2005 16269705 PMC1287661

[B135] Theisen A. R. Murrell J. C. (2005). Facultative methanotrophs revisited. *J. Bacteriol.* 187 4303–4305. 15968038 10.1128/JB.187.13.4303-4305.2005 PMC1151775

[B136] Tischer K. Zeder M. Klug R. Pernthaler J. Schattenhofer M. Harms H. (2012). Fluorescence in situ hybridization (CARD-FISH) of microorganisms in hydrocarbon contaminated aquifer sediment samples. *Syst. Appl. Microbiol.* 35 526–532. 10.1016/j.syapm.2012.01.004 22425347

[B137] Tiwari S. Singh J. S. Singh D. P. (2015). Methanotrophs and CH_4_ sink: effect of human activity and ecological perturbations. *Climate Change Environ. Sustainability* 3 35–50.

[B138] Trotsenko Y. Murrell J. C. (2008). “Metabolic aspects of aerobic obligate methanotrophy,” in *Advances in Applied Microbiology*, eds Laskin A. Sariaslani S. Gadd G. (Amsterdam: Elsevier). 10.1016/S0065-2164(07)00005-6 18395128

[B139] Tsai J. Kratz T. K. Hanson P. C. Wu J. Chang W. B. Arzberger P. W. (2008). Seasonal dynamics, typhoons and the regulation of lake metabolism in a subtropical humic lake. *Freshw. Biol.* 53 1929–1941. 10.1111/j.1365-2427.2008.02017.x

[B140] Tu T. Wu L. Lin Y. Imachi H. Lin L. Wang P. (2017). Microbial community composition and functional capacity in a terrestrial ferruginous, sulfate-depleted mud volcano. *Front. Microbiol.* 8:2137. 10.3389/fmicb.2017.02137 29163423 PMC5673622

[B141] Tuomivirta T. T. Yrjala K. Fritze H. (2009). Quantitative PCR of pmoA using a novel reverse primer correlates with potential methane oxidation in Finnish fen. *Res. Microbiol.* 160 751–756. 10.1016/j.resmic.2009.09.008 19781637

[B142] Urmann K. Lazzaro A. Gandolfi I. Schroth M. H. Zeyer J. (2009). Response of methanotrophic activity and community structure to temperature changes in a diffusive CH_4_ /O2 counter gradient in an unsaturated porous medium. *FEMS Microbiol. Ecol.* 69 202–212. 10.1111/j.1574-6941.2009.00708.x 19496819

[B143] Van Aken B. Peres C. M. Doty S. L. Yoon J. M. Schnoor J. L. (2004). *Methylobacterium populi* sp. nov., a novel aerobic, pink-pigmented, facultatively methylotrophic, methane-utilizing bacterium isolated from poplar trees (Populus x deltoides nigra DN34). *Int. J. Syst. Evol. Microbiol.* 54 1191–1196. 10.1099/ijs.0.02796-0 15280290

[B144] Van Grinsven S. Oswald K. Wehrli B. Jegge C. Zopfi J. Lehmann M. F. (2021). Methane oxidation in the waters of a humics-rich boreal lake stimulated by photosynthesis, nitrite, Fe(III) and humics. *Biogeosciences* 18 3087–3101. 10.5194/bg-2021-3

[B145] Van Teeseling M. C. F. Pol A. Harhangi H. R. van der Zwart S. Jetten M. S. M. Op den Camp H. J. M (2014). Expanding the Verrucomicrobial methanotrophic world: description of three novel species of Methylacidimicrobium gen. nov. *Appl. Environ. Microbiol.* 80 6782–6791. 10.1128/AEM.01838-14 25172849 PMC4249049

[B146] Vorobev A. V. Baani M. Doronina N. V. Brady A. L. Liesack W. Dunfield P. F. (2011). Methyloferulla stellate gen. nov., sp. nov., an acidophilic, obligately methanotrophic bacterium that possesses only a soluble methane monooxygenase. *Int. J. Syst. Evol. Microbiol.* 61 2456–2463. 10.1099/ijs.0.028118-0 21097638

[B147] Vuillemin A. Horn F. Alawi M. Henny C. Wagner D. Crowe S. A. (2017). Preservation and significance of extracellular DNA in ferruginous sediments from Lake Towuti. *Indonesia. Front. Microbiol.* 8:1440. 10.3389/fmicb.2017.01440 28798742 PMC5529349

[B148] Welte C. U. Rasigraf O. Vaksmaa A. Versantvoort W. Arshad A. Op den Camp H. J. (2016). Nitrate- and nitrite-dependent anaerobic oxidation of methane. *Environ. Microbiol. Rep.* 8 941–955. 10.1111/1758-2229.12487 27753265

[B149] West W. E. Creamer K. P. Jones S. E. (2016). Productivity and depth regulate lake contributions to atmospheric methane. *Limnol. Oceanogr.* 61 S51–S61. 10.1007/s11356-020-08296-0 32170622

[B150] Wilson K. (2001). Preparation of genomic dna from bacteria. *Curr. Protoc. Mol. Biol*. 56, 2.4.1–2.4.5. 10.1002/0471142727.mb0204s56 18265184

[B151] Wu X. Xi W. Ye W. Yang H. (2007). Bacterial community composition of a shallow hypertrophic freshwater lake in China, revealed by 16S rRNA gene sequences. *FEMS Microbiol. Ecol.* 61 85–96. 10.1111/j.1574-6941.2007.00326.x 17506827

[B152] Yang P. Lai D. Y. Yang H. Lin Y. Tong C. Hong Y. (2022). Large increase in CH_4_ emission following conversion of coastal marsh to aquaculture ponds caused by changing gas transport pathways. *Water Res.* 222:118882. 10.1016/j.watres.2022.118882 35882096

[B153] Yang S. Liebner S. Alawi M. Ebenhoh O. Wagner D. (2014). Taxonomic database and cut-off value for processing mcrA gene 454 pyrosequencing data by MOTHUR. *J. Microbiol. Methods* 103 3–5. 10.1016/j.mimet.2014.05.006 24858450

[B154] Yang Y. Chen J. Tong T. Li B. He T. Liu Y. (2019). Eutrophication influences methanotrophic activity, abundance and community structure in freshwater lakes. *Sci. Total Environ.* 662 863–872. 10.1016/j.scitotenv.2019.01.307 30708301

[B155] Yoshimizu C. Yoshiyama K. Tayasu I. Koitabashi T. Nagata T. (2010). Vulnerability of a large monomictic lake (Lake Biwa) to warm winter event. *Limnology* 11 233–239. 10.1007/s10201-009-0307-3

[B156] Yvon-Duroucher G. Allen A. P. Bastviken D. Conrad R. Gudasz C. St-Pierre A. (2014). Methane fluxes show consistent temperature dependent across microbial to ecosystem scales. *Nature* 507 488–495. 10.1038/nature13164 24670769

[B157] Zhu B. Karwautz C. Andrei S. Klingl A. Pernthaler J. Lueders T. (2022). A novel *Methylomirabilota* methanotroph potentially couples methane oxidation to iodate reduction. *mLife* 1 323–328. 38818217 10.1002/mlf2.12033 PMC10989891

[B158] Zhu G. Zhou L. Wang Y. Wang S. Guo J. Long X. (2015). Biogeographical distribution of denitrifying anaerobic methane oxidizing bacteria in Chinese wetland ecosystems. *Environ. Microbiol.* 7 128–138. 10.1111/1758-2229.12214 25223900

[B159] Zigah P. K. Oswald K. Brand A. Dinkel C. Wehrli B. Schubert C. J. (2015). Methane oxidation pathways and associated methanotrophic communities in the water column of a tropical lake. *Limnol. Oceanogr.* 60 553–572. 10.1002/lno.10035

